# Association of Economic Policies With Hypertension Management and Control: A Systematic Review

**DOI:** 10.1001/jamahealthforum.2023.5231

**Published:** 2024-02-09

**Authors:** Donglan Zhang, Jun Soo Lee, Lisa M. Pollack, Xiaobei Dong, Joanna M. Taliano, Anand Rajan, Nicole L. Therrien, Sandra L. Jackson, Adebola Popoola, Feijun Luo

**Affiliations:** 1 Center for Population Health and Health Services Research, Department of Foundations of Medicine, New York University Grossman Long Island School of Medicine, Mineola; 2 Department of Population Health, New York University Grossman School of Medicine, New York; 3 Division for Heart Disease and Stroke Prevention, Centers for Disease Control and Prevention, Atlanta, Georgia; 4 Joseph J. Zilber School of Public Health, University of Wisconsin-Milwaukee, Milwaukee; 5 Office of Science Quality and Library Services, Office of Science, Centers for Disease Control and Prevention, Atlanta, Georgia

## Abstract

**Importance:**

Economic policies have the potential to impact management and control of hypertension.

**Objectives:**

To review the evidence on the association between economic policies and hypertension management and control among adults with hypertension in the US.

**Evidence Review:**

A search was carried out of PubMed/MEDLINE, Cochrane Library, Embase, PsycINFO, CINAHL, EconLit, Sociological Abstracts, and Scopus from January 1, 2000, through November 1, 2023. Included were randomized clinical trials, difference-in-differences, and interrupted time series studies that evaluated the association of economic policies with hypertension management. Economic policies were grouped into 3 categories: insurance coverage expansion such as Medicaid expansion, cost sharing in health care such as increased drug copayments, and financial incentives for quality such as pay-for-performance. Antihypertensive treatment was measured as taking antihypertensive medications or medication adherence among those who have a hypertension diagnosis; and hypertension control, measured as blood pressure (BP) lower than  140/90 mm Hg or a reduction in BP. Evidence was extracted and synthesized through dual review of titles, abstracts, full-text articles, study quality, and policy effects.

**Findings:**

In total, 31 articles were included. None of the studies examined economic policies outside of the health care system. Of these, 16 (52%) assessed policies for insurance coverage expansion, 8 (26%) evaluated policies related to patient cost sharing for prescription drugs, and 7 (22%) evaluated financial incentive programs for improving health care quality. Of the 16 studies that evaluated coverage expansion policies, all but 1 found that policies such as Medicare Part D and Medicaid expansion were associated with significant improvement in antihypertensive treatment and BP control. Among the 8 studies that examined patient cost sharing, 4 found that measures such as prior authorization and increased copayments were associated with decreased adherence to antihypertensive medication. Finally, all 7 studies evaluating financial incentives aimed at improving quality found that they were associated with improved antihypertensive treatment and BP control. Overall, most studies had a moderate or low risk of bias in their policy evaluation.

**Conclusions and Relevance:**

The findings of this systematic review suggest that economic policies aimed at expanding insurance coverage or improving health care quality successfully improved medication use and BP control among US adults with hypertension. Future research is needed to investigate the potential effects of non–health care economic policies on hypertension control.

## Key Points

**Question:**

What is the association between economic policies and hypertension management and control in the US?

**Findings:**

In this systematic literature review of 31 studies, 15 of 16 studies assessing insurance coverage expansion and all 7 studies evaluating financial incentives for improving quality of care found that these policies were associated with significant improvement in antihypertensive treatment and blood pressure control. Among 8 studies evaluating patient cost sharing, 4 found that increased copayments were associated with decreased adherence to antihypertensive medication.

**Meaning:**

The expansion of insurance coverage and the implementation of financial incentives to improve quality of care were associated with improved hypertension management and control.

## Teaser

This systematic review examines the evidence on the association between economic policies and hypertension management and control among adults with hypertension in the US.

## Introduction

Hypertension—defined as systolic blood pressure (BP) greater than 130 mm Hg, a diastolic BP greater than 80 mm Hg, or the use of prescription medication to lower BP—affects 48% of all US adults (119.9 million people).^1,2^ Because hypertension is a major risk factor for heart disease and stroke, which are leading causes of death in the US,^3^ hypertension control is a national priority.^4^ However, only about 1 in 4 adults with hypertension have their condition controlled,^2^ and disparities exist based on socioeconomic status, race and ethnicity, and geographic region.^5^ The Surgeon General’s Call to Action to Control Hypertension^6^ seeks to prevent adverse outcomes from uncontrolled hypertension—such as myocardial infarction, heart failure, stroke, kidney disease, pregnancy complications, and cognitive decline—by identifying evidence-based interventions that can be implemented and adapted in diverse settings across the US.

To address the pressing issue of uncontrolled hypertension and its associated risks, it is essential to consider broadscale actions. Economic policies such as minimum wages, unemployment benefits, and Medicaid expansion might be potential interventions to improve hypertension control, by reducing cost-related barriers to health care among people with lower incomes.^4^ Economic policies that address health-related social needs, such as the Supplemental Nutrition Assistance Program (SNAP), might also have an effect on hypertension-related outcomes.^7^ Other economic policies, such as cost control policies or reimbursement models, may have unintended consequences and potentially reduce hypertension control.^8^ However, the evidence regarding the associations between these policies and patients’ adherence to antihypertensive medications, or their ability to control hypertension, is limited and inconclusive. It is important to evaluate such policies’ effects on hypertension control before the policies can be implemented widely. To address this literature gap, we conducted a systematic review to examine the evidence on the associations between (1) federal, state, and local economic policies and (2) hypertension management and control. These review findings will provide insight into the potential benefits and limitations of these policies in improving antihypertensive treatment and BP control for the millions of patients with hypertension in the US.

## Methods

### Search Strategy

The review team, comprising health economists, a health sciences librarian, and content experts (including public policy experts, epidemiologists, program managers, pharmacists, and legal experts), formed in October 2022. We conducted this study in accordance with the Preferred Reporting Items for Systematic Reviews and Meta-analyses (PRISMA) reporting guidelines and registered the study protocol on PROSPERO (CRD42022380838).^9,10^ Ethics review was deemed unnecessary because this study did not collect human participant data. Our central search query aimed to identify evidence published in the past 20 years on the association between economic policies and hypertension management and control.

### Data Sources

We conducted systematic literature searches using these databases: PubMed/MEDLINE, Cochrane Library, Embase, PsycINFO, CINAHL, EconLit, Sociological Abstracts, and Scopus. Our search spanned English-language articles published between January 1, 2000, and November 1, 2023. The librarian (J.M.T.) conducted the searches according to the search strategy outlined in Supplement 1, using defined search terms for 2 themes. The first theme covered US economic policies, such as Medicaid expansion, Medicare Part D, minimum wage laws, unemployment insurance, earned income tax credits, and others. The second theme focused on antihypertensive treatment, defined as the use of antihypertensive medications and medication adherence, as well as hypertension control, measured as BP lower than  140/90 mm Hg (the previous guideline used in most prior literature) or a reduced BP. Our search included patients aged 18 years and older (including pregnant people) with hypertension. The detailed search terms are presented in eTable 1 in Supplement 1. To supplement our searches, 2 investigators (J.L. and A.R.) independently reviewed the reference lists of the selected articles, which yielded 5 additional articles (Figure). The searches and analysis were completed in November 2023.

**Figure.**
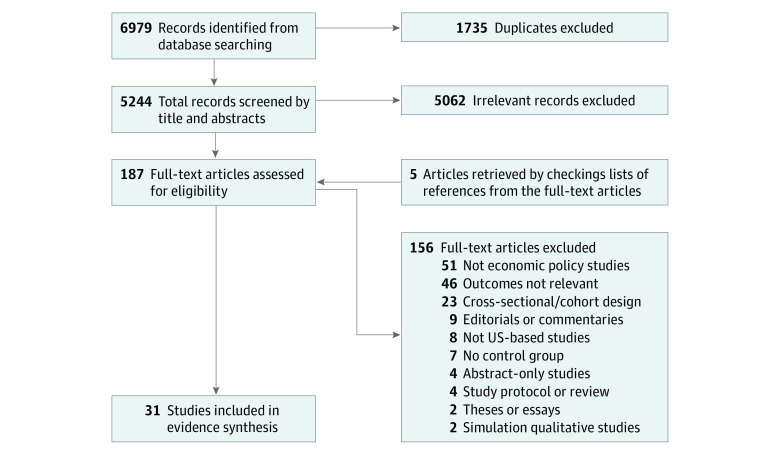
Flowchart of Study Selection Process

### Study Selection

Two investigators (J.L. and A.R.) independently screened titles and abstracts using prespecified inclusion and exclusion criteria (eTable 2 in Supplement 1). Discrepancies were resolved through discussions with third and fourth reviewers (D.Z. and F.L.) to reach a consensus. After retrieving the pertinent full-text articles, 2 investigators (J.L. and A.R.) independently reviewed all the full-text articles and used the Population Intervention Comparison Outcome (PICO) principle to select eligible articles. The PICO principle included these criteria: (1) population: adult patients with hypertension; (2) intervention: evaluation of specified economic policies in the US; (3) comparison: rigorous study design (experimental, eg, randomized clinical trials or quasi-experimental, eg, difference-in-differences and interrupted time series) with a control group (excluding descriptive articles or simulation studies); and (4) outcome: assessment of antihypertensive treatment or BP control. We excluded reports such as essays and dissertations not published in peer-reviewed journals. Any discrepancies in the review of full-text articles were resolved through consensus.

### Data Extraction

For each selected study, 1 investigator (D.Z.) extracted information on the study population, study design, analytical model(s), hypertension-related outcome measures, data source(s), economic policies, comparator, main finding(s), conclusion(s), and funding source(s). A second investigator (F.L.) reviewed the extracted information for completeness, accuracy, and consistency.

### Quality Assessment

Four investigators (X.D., J.L., F.L., and D.Z.) independently assessed the quality of included studies using criteria related to confounding bias, selection bias, measurement bias, and reporting bias. The initial criteria were established by the Agency for Healthcare Research and Quality in their 2017 guidance on assessing the risk of bias in systematic reviews of health care interventions; the criteria were later modified by the review team iteratively to fit the purpose of this study (eTable 3 in Supplement 1 contains details).^11^ Each study was assigned a final quality score on the risk of bias, which was agreed on by the 4 independent reviewers. Quality scores were based on a 10-point scale: low (8-10), moderate (5-7), or high (1-4) risk. Discrepancies were resolved by consensus among the reviewers.

## Results

The literature search resulted in 6979 records, of which 1735 were excluded as duplicates (Figure). After screening the titles and abstracts, we retrieved 187 full-text articles and assessed them against the inclusion criteria. Overall, 31 articles met the inclusion criteria and were included in the systematic review.

### Study Characteristics

The characteristics of the included studies are presented in Table 1. These studies examined various policy interventions. Sixteen studies assessed policies for insurance coverage expansion, including 4 on Medicare Part D^12,13,14,15^ and 12 on Medicaid expansion.^16,17,18,19,20,21,22,23,24,25,26,27^ Eight studies evaluated cost-sharing policies in health care, such as Medicaid prior authorization (n  =  1),^28^ changes in copayment for antihypertensive medications (n  =  3),^29,30,31^ medication coverage gap (n  =  1),^32^ copayment reductions and/or value-based insurance design (VBID, n = 1),^33^ and prescription limit/cap in Medicaid programs (n  =  2).^34,35^ The remaining 7 studies evaluated financial incentive programs for improving quality such as the pay-for-performance (P4P) model,^36^ financial incentives paid to physicians and/or patients,^37,38^ Medicaid Incentives for the Prevention of Chronic Diseases,^39^ and innovative payment models including the Accountable Care Organization (ACO),^40^ patient-centered medical home,^41^ and the Million Hearts Cardiovascular Disease (CVD) Risk Reduction Model programs.^42^ The policies were implemented at different levels: nationwide (n  =  17),^12,13,14,15,18,19,20,21,23,24,25,27,28,32,34,40,42^ state-specific (n  =  10),^16,22,26,29,30,33,35,39,41,43^ or within specific health care settings (n  =  4).^31,36,37,38^ The study populations primarily consisted of Medicaid (n  =  16),^16,18,19,20,21,22,23,24,25,26,27,28,29,30,34,35,39^ and Medicare beneficiaries (n  =  8),^12,13,14,15,32,40,42,44^ with 3 studies including privately insured populations,^33,36,41^ 3 focusing on the US Department of Veterans Affairs (VA),^31,37,38^ and 1 on uninsured populations.^17^ Secondary data sources, including claims databases (n  =  16),^13,14,18,19,20,28,29,31,32,33,34,35,39,40,41,42^ surveys (n  =  7),^12,15,21,23,25,30,44^ and electronic health records (EHRs, n  =  7),^16,22,24,26,36,37,38^ were used for analysis. One study used primary data collection.^17^ Most studies used a difference-in-differences (DiD) approach to assess policy effects (n  =  26),^12,13,14,15,16,18,19,20,21,22,23,24,25,26,27,28,29,30,31,32,33,34,35,39,40,41^ whereas 2 studies used randomized controlled trial (RCT) designs,^17,38^ and 3 studies used a cluster randomized clinical trial design.^36,37,42^ Regarding outcome measures, 15 studies used antihypertensive treatment (6 assessed medication adherence using the proportion of days covered [PDC] measure^14,29,31,32,40,41^),^13,14,15,23,25,27,28,29,31,32,33,34,35,40,41^ 11 studies evaluated BP control,^12,16,18,19,20,21,22,24,26,36,39^ and 5 evaluated both outcomes.^17,30,37,38,42^ Further details on the policy, study population, data sources, study design, and hypertension-related outcome measures for each study are in Supplement 1 (eTable 4).

**Table 1.** Selected Characteristics of the 31 Included Studies

Source	Location		Population	Study design	Data source	Economic policy	IMP, year	Hypertension-related measure
Fischer et al,^28^ 2007	National	Medicaid		Quasi-experimental (DD)	CMS	Prior authorization^b^	2004	% ARB dispensed
Maciejewski et al,^31^ 2010	4 VAMC	VA		Quasi-experimental (DD)	VAMC	Drug copay^b^	2002	Medication adherence
Zhang et al,^14^ 2010	National	Medicare		Quasi-experimental (DD)	CMS	Part D^a^	2006	Medication adherence
Zhang et al,^13^ 2011	National	Medicare		Quasi-experimental (DD)	CMS	Part D^a^	2006	% ARB dispensed
Li et al,^32^ 2012	National	Medicare		Quasi-experimental (DD)	CMS	Coverage gap^b^	2006	Medication adherence
Wang et al,^35^ 2013	LA, IN	Medicaid		Quasi-experimental (DD)	Medicaid	Prescription limit^b^	2003	Medication discontinuation
Baicker et al,^17^ 2013	OR	Uninsured		RCT	Data collection	Expansion^a^	2008	SBP and DBP; an inventory of medications
Bardach et al,^36^ 2013	NYC	Medicaid, uninsured and privately insured		Cluster RCT	EHR	P4P^c^	2009	BP
Petersen et al,^37^ 2013	12 VA clinics	VA		Cluster RCT	EHR	Incentives^c^	2008	Control rate; medication use
Zimmer et al,^15^ 2014	National	Medicare		Quasi-experimental (DD)	MEPS	Part D^a^	2006	No. prescriptions
Hirth et al,^33^ 2016	7 States	Insurance for state employees		Quasi-experimental (DD)	Commercial claims	VBID^b^	2011	Medication adherence
Amin et al,^29^ 2017	NC, GA	Medicaid		Quasi-experimental (DDD)	CMS	Drug copay^b^	2001	Medication adherence
Cole et al,^18^ 2017	National	Medicaid		Quasi-experimental (DD)	HRSA	Expansion^a^	2014	Control rate
Hatch et al,^22^ 2017	OR	Medicaid		Quasi-experimental (DD)	OCHIN	Expansion^a^	2008	Control rate and BP
McWilliams et al,^40^ 2017	National	Medicare		Quasi-experimental (DD)	CMS	ACO^c^	2012	Medication adherence
Kostova et al,^30^ 2017	18 States	Medicaid		Quasi-experimental (DR)	NHANES	Drug copay^b^	2010	Uncontrolled rate; self-reported medication use
Adams et al,^34^ 2017	National	Dual-eligibles with cancer		Quasi-experimental (ITS)	CMS	Drug caps^b^	2005	Prevalence of medication use
Cole et al,^20^ 2018	National	Medicaid		Quasi-experimental (DD)	HRSA	Expansion^a^	2014	Control rate
Diebold et al,^12^ 2018	National	Medicare		Quasi-experimental (DD)	HRS	Part D^a^	2006	Better/worse control
Romaire et al,^39^ 2018	6 States (NS)	Medicaid		Quasi-experimental (DD)	Medicaid	MIPCD^c^	2013	Control rate
Kaboli et al,^38^ 2018	13 VA clinics	VA		RCT	EHR	Incentives^c^	2008	Thiazide prescribing; BP
Angier et al,^16^ 2020	CA, OH, OR, WA, WI	Medicaid		Quasi-experimental (DD)	ADVANCE EHR	Expansion^a^	2014	Control rate
Margerison et al,^25^ 2020	National	Medicaid		Quasi-experimental (DD)	BRFSS	Expansion^a^	2014	Self-reported medication use
Marino et al,^26^ 2020	CA, HI, MD, MN, NM, OH, OR, RI, WA, WI	Medicaid		Quasi-experimental (DD)	ADVANCE EHR	Expansion^a^	2014	SBP and DBP
Cole et al,^19^ 2021	National	Medicaid		Quasi-experimental (DD)	HRSA	Expansion^a^	2014	Control rate
Gotanda et al,^21^ 2021	National	Medicaid		Quasi-experimental (DD)	NHANES	Expansion^a^	2014	SBP and DBP
Kim et al,^23^ 2021	National	Medicaid		Quasi-experimental (DD)	BRFSS	Expansion^a^	2014	Self-reported medication use
Lanese et al,^24^ 2021	National	Medicaid		Quasi-experimental (DD)	NHCHC	Expansion^a^	2014	Control rate
Peterson et al,^42^ 2021	National	Medicare		Cluster RCT	CMS	Million Hearts^c^	2017	Drug initiation and BP
Sumarsono et al,^27^ 2021	National	Medicaid		Quasi-experimental (DD)	MSDUD & CPS	Expansion^a^	2014	Prescription rate
Fakeye et al,^41^ 2022	MD	Medicaid and privately insured		Quasi-experimental (DD)	MMPP	PCMH^c^	2011	Medication adherence

Abbreviations: ACO, accountable care organization; ADVANCE, Accelerating Data Value Across a National Community Health Center Network; ARB, angiotensin receptor blockers; BP, blood pressure; BRFSS, Behavioral Risk Factor Surveillance System; CMS, Centers for Medicare & Medicaid Services; CPS, current population survey; DBP, diastolic blood pressure; DD, difference-in-differences; DDD, difference-in-difference-in-differences; DR, differencing regression; EHR, electronic health records; HRS, Health and Retirement Study; HRSA, Health Resources and Services Administration; IMP, implementation; ITS, interrupted time series; MEPS, Medical Expenditures Panel Survey; MIPCD, Medicaid Incentives for Prevention of Chronic Diseases; MMPP, Maryland Multi-Payor Patient-Centered Medical Home Program; MSDUD, Medicaid State Drug Utilization Data set; NHANES, National Health and Nutrition Examination Survey; NHCHC, National Health Care for the Homeless Council; NS, not specified; OCHIN, a nonprofit network of community health centers; P4P, pay-for-performance; PCMH, patient-centered medical home; RCT, randomized clinical trial; SBP, systolic blood pressure; VAMC, Veterans Affairs Medical Center; VBID, value-based insurance design.

^a^ Insurance coverage expansion (eg, Medicaid expansion, Medicare Part D coverage).

^b^ Cost sharing in health care (eg, increases in patient cost sharing for prescription drugs, copayment reductions, or value-based insurance design).

^c^ Financial incentives for quality (eg, pay-for-performance, financial incentives, or alternative payment models aimed at improving the quality of care).

### Associations Between Insurance Coverage Expansion and BP Control

Among the 16 studies evaluating insurance coverage expansion policies, most found a statistically significant positive association with hypertension management and control (Table 2).^12,13,14,15,16,18,19,20,21,22,23,24,25,26^ The outcome measures varied, with 6 studies measuring antihypertensive treatment as an outcome,^13,14,15,23,25,44^ and 10 measuring BP control as an outcome.^12,16,17,18,19,20,21,22,24,26^ For Medicare Part D expansion, 4 studies found a statistically significant increase in the number of antihypertensives prescribed, filled, or taken, and with better BP control.^12,13,14,15^ For Medicaid expansion, 1 study reported no significant association with BP control after 1 year of implementation.^17^ The other 10 studies consistently showed a positive association with antihypertensive treatment or BP control.^16,18,19,20,22,23,24,25,26,27^ One study found a significant reduction in systolic BP, but no significant association with diastolic BP.^21^

**Table 2.** Association Between Economic Policies and Hypertension Management and Control

Type and source	Economic policies	No.	Main findings	
			Outcome measurement	Estimates
Insurance coverage expansion				
Zhang et al,^14^ 2010	Medicare Part D	T: n = 418; C: n = 3027	No. of antihypertensive pills taken per day of treatment	DD = 0.22 (95% CI, 0.16-0.28)
Zhang et al,^13^ 2011	Medicare Part D	T: n = 1478; C: n = 4253	Average daily counts of any antihypertensive filled each year	OR = 1.40 (95% CI, 1.25-1.56)
Baicker et al,^14^ 2013	Medicaid expansion	T: n = 10 405; C: n = 10 340	Mean SBP and DBP; current use of antihypertensive medications	No difference
Zimmer et al,^15^ 2014	Medicare Part D	T: n = 15 133; C: n = 21 008	No. of antihypertensives prescribed per senior per year	11% (*P* < .05)
Cole et al,^18^ 2017	Medicaid expansion	T: n = 492 (CHCs); C: n = 565 (CHCs)	Hypertension control rate (BP <140/90 mm Hg) for each CHC	2.1 (95% CI, 0.2-4.0)
Hatch et al,^22^ 2017	Medicaid expansion in Oregon	T: n = 622; C: n = 622	Time from uncontrolled hypertension to a controlled hypertension	HR = 1.35 (*P* < .001)
Cole et al,^20^ 2018	Medicaid expansion	T: n = 578 (CHCs); C: n = 431 (CHCs)	Hypertension control rate for each CHC	2.1% (95% CI, 0.2-4.0)
Diebold et al,^12^ 2018	Medicare Part D	T: n = 536; C: n = 1172	HBPUC is better, about the same, or worse than it was in the previous wave	0.59 (*P* < .05)
Angier et al,^16^ 2020	Medicaid expansion in 5 states	T: n = 3054; C: n = 2264	Controlled hypertension defined as whether a patient’s BP was <140/90 mm Hg	T: 8.6% (*P* < .05); C: 0.9%
Margerison et al,^25^ 2020	Medicaid expansion	T: n = 16 499; C: n = 41 866	Self-reported measure of BP medication currently taken	7.9% (95% CI, 3.1-12.8)
Marino et al,^26^ 2020	Medicaid expansion in 10 states	T: n = 2483; C: n = 2888	Mean SBP and DBP	SBP: −1.76 (95% CI, −1.34 to −2.19); DBP: −1.04 (95% CI, −0.77 to −1.30)
Cole et al,^19^ 2021	Medicaid expansion	T: n = 578 FQHCs; C: n = 368 FQHCs	Proportion of patients with hypertension with a BP <140/90 mm Hg	1.61% (95% CI, 0.58-2.64); by year 5, 2.36% (95% CI, 1.01-3.71)
Gotanda et al,^21^ 2021	Medicaid expansion	T: n = 4232; C: n = 1869	Mean SBP and DBP	SBP: −3.03 (95% CI, −5.33 to −0.73); DBP: no difference
Lanese et al,^24^ 2021	Medicaid expansion	T: n = 31 (states); C: n = 20 (states)	Patients with hypertension with a BP <140/90 mm Hg	3.68% (*P* < .001)
Kim et al,^23^ 2021	Medicaid expansion	T: n = 17 (states); C: n = 19 (states)	The length of time since the patients last took their BP medicine	0.70% (*P* < .001)
Sumarsono et al,^27^ 2021	Medicaid expansion	T: n = 29 541 647; C: n = 20 334 618	No. of filled antihypertensive prescriptions per 1000 people quarterly	63.2 (95% CI, 47.3-79.1)
Cost-sharing in health care				
Fischer et al,^28^ 2007	Medicaid PA	T: n = 19 (states); C: n = 18 (states)	Proportion of RAAS-blocking-agent total units dispensed accounted for by ARBs	−0.4%
Maciejewski et al,^31^ 2010	Drug copay in VA	T: n = 3545; C: n = 3545	Adherence to antihypertensive medications measured by PDC	−1.8% (95% CI, −1.8 to −1.9)
Li et al,^32^ 2012	Medicare drug coverage gap	T: n = 22 251; C: n = 39 528	Monthly No. of antihypertensive prescriptions per patient	−4.8% (*P* < .05)
Wang et al,^35^ 2013	Medicaid monthly prescription limit	T: n = 2525; C: n = 1700	Discontinuation of therapy measured as patients without medication for ≥30 d	No difference
Hirth et al,^33^ 2016	Value-based insurance design	T: n = 64 165; C: n = 215 314	Medication adherence measured by MPR	Thiazide 0.5 (NS) Y1, 0.6 (NS) Y2; ACEi 1.0 (*P* < .10) Y1, 1.0 (*P* < .05) Y2; ARB 0.5 Y1, 0.8 (*P* < .05) Y2
Amin et al,^29^ 2017	Medicaid drug copay for brand-name medications	T: n = 22 596; C: n = 35 554	Percentage of fully adherent enrollees (PDC ≥80%) decreased adherence	−1.2% (95% CI, −0.02 to −0.01)
Kostova et al,^30^ 2017	Medicaid drug copayment	T: n = 1128; C: n = 7872	Uncontrolled hypertension rate; self-reported use of antihypertensive drugs	7.7 (*P* < .001); −2.4
Adams et al,^34^ 2017	Transition from Medicaid drug caps to Medicare Part D	T: n = 196; C: n = 642	Antihypertensive days of supply; differences between Black and White patients in trend for days supply	8.6 (95% CI, 1.22 to 16.0); −1.83 (95% CI, −2.81 to −0.85)
Financial incentives for quality				
Bardach et al,^36^ 2013	Pay-for-performance	T: n = 42 (clinics); C: n = 42 (clinics)	Proportion of patients with hypertension with a BP <140/90 mm Hg	5.5% (95% CI, 1.6%-9.3%)
Petersen et al,^37^ 2013	Financial incentives	T: 1 n = 19 (physicians) 2 n = 20 (physicians); 3 n = 19 (physicians); C: n = 19 (physicians)	Proportion of patients achieving BP control or appropriate response to uncontrolled BP; proportion of medication adjustment	8.36% (95% CI, 2.40%-13.00%); 15.36% (95% CI, 0.20%-28.41%)
McWilliams et al,^40^ 2017	Medicare ACO	T: n = 2 690 607; C: n = 8 158 617	Medication adherence measured by mean PDC	0.4% (*P* = .003)
Romaire et al,^39^ 2018	MIPCD	T: n = 244; C: n = 220	Patients with hypertension with an SBP<140 mm Hg	OR = 2.10 (95% CI, 1.01-4.35)
Kaboli et al,^38^ 2018	Patient education and financial incentives	T: 1 n = 143; 2 n = 128; 3 n = 131; C: n = 196	Thiazide prescribing and BP control	Group 1, 2, 3 vs C: 24.5%, 25.8%, 32.8% and 9.7% (*P* < .001); 3 vs C: OR = 1.73 (95% CI, 1.06-2.83)
Peterson et al,^42^ 2021	Medicare Million Hearts Cardiovascular risk reduction program	T: n = 169 (POs); C: n = 161 (POs)	Whether patients with clinical risk factors initiated or intensified antihypertensive therapy and SBP	2.5% (95% CI, 0.9-4.2); SBP: −1.7 (95% CI, −2.8 to −0.6)
Fakeye et al,^41^ 2022	Medicaid and privately insured PCMH	T: n = 51(MP-PCMHs); C: n = 51(SP-PCMHs)	Medication adherence measured by the semiannual medication possession ratio	2.05% (95% CI, 0.72-3.37) by year 3; 1.67% (95% CI, 0.32-3.01) by year 5

Abbreviations: ACO, accountable care organization; ARB, angiotensin receptor blocker; BP, blood pressure; CHCs, community health centers; DBP, diastolic blood pressure; DD, difference-in-difference; FQHCs, Federally Qualified Health Centers; HBPUC, high blood pressure under control; HR, hazard ratio; MIPCD, Medicaid incentives for prevention of chronic diseases; MP-PCMH, multipayer patient-centered medical home; MPR, medication possession ratios; OR, odds ratio; PA, prior authorization; PDC, proportion of days covered; POs, provider organizations; RAAS, renin-angiotensin-aldosterone system; SBP, systolic blood pressure; SP-PCMHs, single-payer patient-centered medical home; VA, Veterans Affairs.

### Associations Between Cost-Sharing Policies and BP Control

Among the 8 studies evaluating cost-sharing policies in Medicaid, Medicare, or VA programs (Table 2), 2 studies evaluated copayment reduction effects.^33,34^ One revealed increased medication adherence with reduced copays for essential drugs in Connecticut state employees’ insurance.^33^ The second study showed a transition from a capped Medicaid drug program to a noncapped Medicare program, resulting in an 8.6% improvement in antihypertensive days of supply and a 1.83% reduction in the difference between White and Black individuals.^34^ Of the remaining 6 studies that evaluated increased copayments or restrictions on drug spending, 2 studies on Medicaid prior authorization and monthly prescription limit showed no significant effect on use of antihypertensive medication.^28,35^ The other 4 studies yielded differing results.^29,30,31,32^ One study in VA medical centers showed that increased drug copay rates were associated with a significant reduction (1.8%) in adherence to antihypertensive medications.^31^ Another study revealed that the implementation of Medicaid drug copayments associated with a 7.7–percentage point increase in the prevalence of uncontrolled hypertension.^30^ Furthermore, another study found that patients entering the Medicare Part D coverage gap experienced a decrease in monthly antihypertensive prescriptions (4.8%).^32^ Finally, 1 study observed that Medicaid copayment increased for brand-name medications and reduced prescription supply were associated with a statistically significant 1.2% reduction in the percentage of enrollees who were adherent to all antihypertension medications (PDC ≥ 80%).^29^

### Associations Between Financial Incentives for Quality Policies and BP Control

Seven studies assessed financial incentives for quality policies and all showed a significant positive association with antihypertensive treatment and BP control (Table 2). One study found that a P4P program implemented in the clinic improved BP control by 5.5%.^36^ In addition, 2 studies^37,38^ using financial incentives in VA clinics demonstrated significant enhancement in BP control and antihypertensive prescriptions. Another study found that Medicare ACOs were associated with improved medication adherence,^40^ and another study on Medicaid financial incentive programs (ie, New York’s process-only incentives provided to patients with Medicaid who attended clinical visits and filled/refilled prescriptions) demonstrated a significant association between financial incentives and improved BP control (odds ratio, 2.10).^39^ Medicare’s Million Hearts CVD Risk Reduction Model showed increased medication use and a significant reduction in systolic BP among patients seen by contracted provider organizations.^42^ Lastly, a study comparing single-payer and multipayer patient-centered medical homes found that the multipayer approach significantly improved medication adherence in years 3 and 5 after policy implementation.^41^

### Bias Assessment

Table 3 summarizes the risk of bias assessment results. Among the included studies, 10 (32%) had a low risk of bias, with an overall quality score of 8 to 10,^17,18,19,21,26,30,36,37,38,42^ whereas 18 (58%) had a moderate risk of bias, scoring 5 to 7.^12,13,14,16,20,22,25,27,28,29,31,32,33,34,35,39,40,41^ Only 3 studies were classified as having a high risk of bias.^15,23,24^ Regarding specific bias domains, 17 studies adequately controlled for confounding bias, including having individual-level, contextual-level (eg, state, clinic), and time controls,^16,17,18,19,20,21,22,25,26,30,32,36,37,38,40,41,42^ whereas 4 studies did not adequately control for confounding bias.^12,15,24,28^ Six studies had a low risk of selection bias due to their RCT or cluster RCT designs and appropriately addressed attrition issues,^17,18,36,37,38,39^ whereas the remaining articles selected study participants that did not reflect the target population, or they did not address missing attrition issues adequately. Regarding outcome measurement, 2 studies used actual BP readings,^17,21^ whereas the rest relied on claims or EHR or self-reported data to estimate the outcomes. Twenty-two studies disclosed funding sources and potential conflicts of interest,^12,14,16,17,19,20,21,22,24,25,26,30,33,34,35,36,37,38,39,41,42,43^ whereas the remaining studies may have had potential conflicts of interest because investigators received payments from the pharmaceutical industry or insurance companies.

**Table 3.** Risk of Bias Assessment Results

Source	Risk of bias due to confounding (0-3)^a^	Risk of bias due to selection (0-2)	Risk of bias in measurement of outcomes (1-3)	Risk of bias in reported results (0-2)	Overall study quality (1-10)	Overall study quality metrics^b^
Fischer et al,^28^ 2007	1	1	2	1	5	Moderate risk
Maciejewski et al,^31^ 2010	2	1	2	1	6	Moderate risk
Zhang et al,^14^ 2010	2	0	2	1	5	Moderate risk
Zhang et al,^13^ 2011	2	0	2	2	6	Moderate risk
Li et al,^32^ 2012	3	1	2	1	7	Moderate risk
Wang et al,^35^ 2013	2	1	2	2	7	Moderate risk
Baicker et al,^17^ 2013	3	2	3	2	10	Low risk
Bardach et al,^36^ 2013	3	2	2	2	9	Low risk
Petersen et al,^37^ 2013	3	2	2	2	9	Low risk
Zimmer et al,^15^ 2014	1	1	1	1	4	High risk
Hirth et al,^33^ 2016	2	1	2	2	7	Moderate risk
Amin et al,^29^ 2017	2	1	2	1	6	Moderate risk
Cole et al,^18^ 2017	3	2	2	1	8	Low risk
Hatch et al,^22^ 2017	3	0	2	2	7	Moderate risk
McWilliams et al,^40^ 2017	3	1	2	1	7	Moderate risk
Kostova et al,^30^ 2017	3	1	2	2	8	Low risk
Adams et al,^34^ 2017	2	1	2	2	7	Moderate risk
Cole et al,^20^ 2018	3	0	1	2	6	Moderate risk
Diebold et al,^12^ 2018	1	1	1	2	5	Moderate risk
Romaire et al,^39^ 2018	2	2	1	2	7	Moderate risk
Kaboli et al,^38^ 2018	3	2	2	2	9	Low risk
Angier et al,^16^ 2020	3	0	2	2	7	Moderate risk
Margerison et al,^25^ 2020	3	0	1	2	6	Moderate risk
Marino et al,^26^ 2020	3	1	2	2	8	Low risk
Cole et al,^19^ 2021	3	1	2	2	8	Low risk
Gotanda et al,^21^ 2021	3	1	3	2	9	Low risk
Kim et al,^23^ 2021	2	0	1	1	4	High risk
Lanese et al,^24^ 2021	0	0	2	2	4	High risk
Peterson et al,^42^ 2021	3	1	2	2	8	Low risk
Sumarsono et al,^27^ 2021	2	1	2	2	7	Moderate risk
Fakeye et al,^41^ 2022	3	0	2	2	7	Moderate risk

^a^ Representing the score range. The maximum score for risk of bias due to confounding and in the measurement of outcomes is 3. The maximum score for risk of bias due to selection and in reported results is 2. The maximum score for overall study quality is 10. A higher score implies a lower risk of bias.

^b^ Overall study quality score: 8 to 10 low risk; 5 to 7 moderate risk; 1 to 4 high risk.

## Discussion

This review is the first to our knowledge to critically assess the associations between economic policies and hypertension management and control in the US over the past 20 years. We identified and analyzed 31 empirical studies. These studies focused on 3 categories of economic tools: insurance coverage expansion,^12,13,14,15,16,17,18,19,20,21,22,23,24,25,26,27^ cost sharing in health care,^28,29,30,31,32,33,34,35^ and financial incentives for quality.^36,37,38,39,40,41,42^

Results of this analysis revealed that expanding insurance coverage through policies such as Medicaid expansion was positively associated with improved hypertension management and control. Eleven studies reported that Medicaid expansion increased medication use,^23,25,27^ and better BP control,^16,18,19,20,21,22,24,26^ with varying effect sizes; however, the landmark study, the Oregon Health Insurance Experiment, did not observe a significant effect on BP control.^17^ The variations in results could be attributed to factors such as the study population (nationwide^18,20,25^ vs state^16,17^) and years after implementation (1 year^17^ vs several years^22,26,27^). Due to the heterogeneity among policies and study populations, a meta-analysis was not feasible.

The evidence on the association between Medicare Part D coverage expansion and hypertension management was consistent.^12,13,14,15^ Three studies reported improved antihypertensive medication use,^13,14,15^ and 1 study^12^ reported improved BP control. These results suggested that Medicare drug policies, under the Affordable Care Act and more recently in the Inflation Reduction Act,^45^ aimed to close the drug coverage gaps and improve affordable access to antihypertensive medications for beneficiaries, can effectively reduce the burden of uncontrolled hypertension.^46^

The present analysis found a consistent and positive association between financial incentives policies and hypertension treatment.^36,37,38,39,40,41,42^ Seven studies on financial incentives to improve targeted quality indicators—regarding P4P,^36^ ACOs,^40^ incentives to promote preventive services,^37,38,39^ patient-centered medical homes,^41^ and the Million Hearts CVD Risk Reduction Model^42^—demonstrated improved medication adherence^37,40,41,42^ and reduced BP.^36,37,38,39,42^ These programs align financial incentives with better patient outcomes, promote coordinated care, encourage collaboration among health care professionals, and prioritize preventive care and chronic disease management.^47^ Although such programs are often implemented locally, these findings suggest that targeted policies and innovative payment and delivery approaches could contribute to achieving the US Surgeon General’s goal of improving hypertension management.^4^

We identified 8 studies on cost-sharing policies.^28,29,30,31,32,33,34,35^ Four studies found that the policies had a significant negative association with antihypertension medication use,^29,31,32,43^ and 2 studies reported no association with the use of antihypertensive medications.^28,35^ The assessed policies included Medicaid prior authorization,^43^ monthly prescription limits,^35^ and increasing copayments on antihypertensive drugs.^29,31^ In contrast, 2 studies evaluating copayment reduction showed positive association with drug supply or adherence.^33,34^ Prior authorization restricts access to certain medications,^48^ whereas monthly prescription limits impose restrictions on the allowed amount of medication per month (eg, the Louisiana Medicaid program restricted patients to receiving 8 prescriptions per month).^35^ Imposing copayments on antihypertensive drugs may discourage their use but can increase patient costs and reduce medication adherence.^29^ In response to concerns about the policy’s potential adverse effects on hypertension control, the Centers for Medicare & Medicaid Services has introduced a new rule proposal. The primary goal of the proposal is to enhance patient and clinician access to health information while streamlining the prior authorization process and promoting efficiency and transparency.^49^ These changes may improve hypertension treatment.

Although we searched for policies outside of the US health care system, such as SNAP or unemployment insurance, our systematic review did not find any studies meeting our inclusion criteria. We excluded cross-sectional studies and those using self-reported ever diagnosed hypertension as the outcome measure for evaluation. However, some cross-sectional analyses not included in our review have suggested a potential association between other economic policies (such as participation in SNAP and increased paid sick leave) with better hypertension control.^7,50,51,52,53^ Two recent articles^54,55^ using difference-in-differences approaches design revealed a potential association between higher state minimum wages and reduced prevalence of hypertension, as assessed via self-reported hypertension diagnosis. Nevertheless, these studies did not meet our inclusion criteria, highlighting a gap in the literature regarding the effect of a broad range of economic policies on hypertension control.

Previous systematic reviews have assessed the effect of the ACA, and found that Medicaid expansion improved various outcomes, such as insurance coverage,^56^ service use,^57,58^ quality of care,^57^ and reduced racial and ethnic– and income-related disparities.^59^ However, these reviews did not specifically examine the effects of Medicaid expansion on use of antihypertensive medications or BP control outcomes. In contrast, our review focused specifically on hypertension treatment and control, revealing evidence of improved hypertension outcomes after the 2014 Medicaid expansion.^18,19,20,22,23,24,25,26,27,28,29,30,31,32,33,34,35,36,37,38,39,40,41,42,43,44,45,46,47,48,49,50,51,52,53,54,55,56,57^ Previous systematic reviews have explored VBID and P4P models.^60,61^ However, many studies in these reviews did not focus on economic policies (eg, disease management programs run by a hospital or private payers),^62,63^ or were conducted in different countries (eg, P4P in the United Kingdom).^61,64^ This review specifically examined policy-effect studies related to US government-run initiatives, such as Medicaid, Medicare, and VA payment policies. Moreover, most of the studies (90%) had a low or moderate risk of bias. This high-quality evidence is a strength compared with previous systematic reviews, which may have included less rigorous policy analyses.

### Limitations

This review has several limitations. First, the associations found between economic policies and hypertension outcomes in 7 studies were based on self-reported measures of medication use or BP, which can be influenced by measurement error and recall bias.^65^ Objective measures using pharmacy claims or electronic health records were used in 22 studies, providing more reliable data on medication adherence and BP. However, these methods assess only medication acquisition and possession, not actual use. The use of clinically validated BP measurement devices was limited to 2 studies.^17,21^ Second, the review primarily examined short-term policy effects because most studies evaluated policies within a 3-year time frame, thus potentially underestimating long-term effects. Third, the evidence on financial incentive policies for quality stems mainly from local studies, limiting generalizability at a national level.^39,40,41,42^ Fourth, further research is needed to explore the effects of economic policies, beyond health care, on hypertension outcomes. Finally, although many economic policies aim to benefit low-income populations and have the potential to reduce health disparities (eg, Women, Infants, and Children [WIC], Medicaid expansion, SNAP), only 1 study included in our analysis examined the effects of Medicaid drug caps on White-Black differences in antihypertensive drug supply,^34^ indicating a gap in the literature. None of the included studies addressed other racial and ethnic minority populations or other types of disparities such as geographic disparities, where rural communities face a disproportionately higher burden of hypertension.^66^

## Conclusions

The findings of this review highlight the potential benefits and limitations of economic policies in improving hypertension management for the millions of patients in the US at risk for CVD. Policymakers may consider implementing evidence-informed economic policies to address health disparities among patients with hypertension and CVD. Further research is needed to identify effective policy interventions, beyond health care, and for different populations and regions.

## Notes

Supplement 1. eTable 1. Search Terms and Definitions eTable 2. Inclusion and Exclusion Criteria eTable 3. Bias Assessment Checklist and Rubric eTable 4. Evidence Extraction Main Table

Supplement 2. Data Sharing Statement
