# Supplementary material for: Association of Economic Policies With Hypertension Management and Control: A Systematic Review
Source: JAMA Health Forum. 2024 Feb 9;5(2):e235231. doi: 10.1001/jamahealthforum.2023.5231 (PMC10858400; doi:10.1001/jamahealthforum.2023.5231)
Supplement: Supplement 1. — eTable 1. Search Terms and Definitions eTable 2. Inclusion and Exclusion Criteria eTable 3. Bias Assessment Checklist and Rubric eTable 4. Evidence Extraction Main Table [file jamahealthforum-e235231-s001.pdf]

Supplemental Online Content

Zhang D, Lee J, Pollack L, et al. Association of economic policies with hypertension management and control: a systematic review. *JAMA Health Forum*. 2024;5(2):e235231. doi:10.1001/jamahealthforum.2023.5231

eTable 1. Search Terms and Definitions

eTable 2. Inclusion and Exclusion Criteria

eTable 3. Bias Assessment Checklist and Rubric

eTable 4. Evidence Extraction Main Table

This supplemental material has been provided by the authors to give readers additional information about their work.

© 2024 Zhang et al. *JAMA Health Forum.*

eTable 1. Search Terms and Definitions

Concepts	Synonyms/Acronyms
**Economic Policies [a,b]**	
Minimum Wage	"Minimum Wage"
Unemployment Insurance	"Unemployment Insurance" OR "unemployment benefits"
Earned Income Tax Credit	"Earned Income Tax Credit" OR EITC
Temporary Assistance for Needy Families	"Temporary Assistance for Needy Families" OR TANF OR "Aid to Families with Dependent Children" OR "AFDC"
Paid leave	"Paid Family and Medical Leave" OR "Paid Family Leave" OR "Paid Maternity Leave" OR "Paid leave" OR "Sick leave"
Unpaid leave	"Family and Medical leave act" OR FMLA
Affordable Care Act	"Patient Protection and Affordable Care Act" OR "ACA" OR "Affordable Care Act" OR "Obamacare"
Medicaid expansion	"Medicaid Expansion"
Prescription drugs	"Medicare Part D" OR "Medicare Drug Coverage" OR "Medicare prescription drug benefit" OR "Cap on Prescription Drug" OR "Inflation Reduction Act"
Telehealth	"All-Payer Telemedicine Parity" OR "Medicaid reimbursement for audio-only services" OR "Medicare Expansion of Telehealth with 1135 Waiver"
Housing policy	"Eviction Moratoria" OR "Eviction Protection" OR "Housing Policy" OR "Mortgage Relief" OR "Foreclosure Relief" OR "Making Home Affordable"
Food policy: nutrition assistance	"Supplemental Nutrition Assistance Program" OR SNAP OR "emergency supplementary" OR "Meal Replacement Benefits" OR "Nutrition Assistance" OR "Women, Infants, and Children" OR WIC
Economic relief	"Economic Impact Payments" OR "Stimulus Checks" OR "Inflation Relief Checks" OR "Child Tax Credit" OR "American Rescue Plan"
Social Security	"Supplemental Security Income" OR SSI
Medicare Savings Programs	"Medicare Savings Programs" OR "MSP" OR "Qualified Medicare Beneficiary Program" OR QMB OR "Specified Low-Income Medicare Beneficiary Program" OR SLMB OR "Qualifying Individual Program" OR QI OR "Qualified Disabled Working Individual Program" OR QDWI
Prescription drugs: low-income subsidy	"Medicare Part D Low-Income Subsidy" OR "Medicare Part D LIS" OR "Part D Low-Income Subsidy" OR "LIS/Extra Help" OR "State Pharmaceutical Assistance Programs" OR "SPAP" OR "SPAPs" OR "Medicaid Drug Cap"
Reimbursement Policy, Financial incentive	"Reimbursement, Incentive" OR "financial incentive" OR "economic incentive" OR "monetary incentive" OR "pay-for-performance" OR "P4P"
Value-Based Health Insurance Design	"Cost Sharing" OR "Value-Based Health Insurance" OR "Value-Based Purchasing" OR "decrease/ reduce/ eliminate copayment OR co-pay" OR "value-based insurance" OR "value-based health insurance" OR

© 2024 Zhang et al. *JAMA Health Forum.*

	“value-based pricing” OR “value-based benefit” OR “VBBD” OR “VBID” OR “full prescription coverage”
General search term	“economic policy” OR “economic policies”
**Hypertension management and control** [b]	
Hypertension	Hypertension OR "Hypertension"[Mesh] OR “High Blood Pressure” OR "Comorbidity"[Mesh] OR comorbidity OR comorbidities OR “co-morbidity” OR “co-morbidities” OR "Cardiovascular Diseases"[Mesh] OR "Cardiovascular Diseases" OR "Cardiovascular Disease" OR “Antihypertensive Agents” OR “Angiotensin-Converting Enzyme Inhibitors” OR “antihypertensive” OR “anti-hypertensive” OR “beta blocker” OR “diuretic” OR “alpha blocker” OR “vasodilator” OR “ace inhibitor”
**US-based**[c]	
United States	“United States” OR “US” OR “U.S.”

[a] OR all the Economic Policies.
[b] AND Economic Policies and Hypertension concepts.
[c] Because these policies are all US-based, we may not need the “US-based” concept. Including it may limit the search, and we can easily tease out non-US-based studies.

eTable 1. (Cont.) First Round of Literature Search Results (1/1/2000-11/1/2022)

Search Strategy:

Database	Strategy	Run Date	Records
**Medline (OVID) 1946-**	1. Exp Hypertension/ OR (hypertension OR hypertensive OR blood pressure).ti,ab,kf.<br>2. exp "Salaries and Fringe Benefits"/ OR (minimum wage* OR hourly wage* OR hourly salary).ti,ab,kf.<br>3. (Insurance coverage/ AND (Unemployment/ OR (unemploy* OR ((job OR employ*) ADJ2 loss) OR employment status).ti,ab,kf.)) OR (unemployment insurance OR unemployment benefit* OR unemployment coverage).ti,ab,kf.<br>4. (Earned Income Tax Credit OR EITC).ti,ab,kf.<br>5. Exp Public Assistance/ OR Aid to Families with Dependent Children/ OR ("Temporary Assistance for Needy Families" OR TANF OR "Aid to Families with Dependent Children" OR AFDC).ti,ab,kf.<br>6. Sick Leave/ OR (Paid leave OR Sick leave).ti,ab,kf.	11/01/2022	2127

© 2024 Zhang et al. *JAMA Health Forum.*

	7. Family Leave/ OR ("Family and Medical Leave Act" OR FMLA OR family leave OR medical leave).ti,ab,kf.<br>8. "Patient Protection and Affordable Care Act"/ OR (ACA OR Affordable Care Act OR Obamacare).ti,ab,kf.<br>9. "Centers for Medicare and Medicaid Services, U.S."/ OR Medicaid/ OR Dual MEDICAID MEDICARE Eligibility/ OR Medicaid Expansion.ti,ab,kf.<br>10. Exp Medicare/ OR Medicare Part D/ OR ("Part D" OR (Medicare AND (drug coverage OR drug insurance OR drug benefit* OR (prescription* ADJ2 cost*))) OR (cap ADJ5 cost*) OR "Inflation Reduction Act" OR (medicare ADJ5 (savings OR beneficiary OR fee-for-service*)) OR "Qualifying Individual Program" OR "Qualified Disabled Working Individual Program").ti,ab,kf.<br>11. "All-Payer Telemedicine Parity" OR "Medicaid reimbursement for audio-only services" OR "Medicare Expansion of Telehealth with 1135 Waiver" OR ((telehealth OR telemedicine OR tele-health OR tele-medicine OR ehealth OR mhealth OR mobile health OR audio only service*) ADJ5 (parity OR polic* OR law* OR regulation* OR payment* OR payer OR all-payer OR reimburse* OR waiver*)).ti,ab,kf,hw.<br>12. (Housing/ AND Public Policy/) OR (Eviction Moratori* OR Eviction Protection* OR Housing Polic* OR Mortgage Relief OR Foreclosure Relief OR ((Home* OR housing) ADJ2 Afford*)).ti,ab,kf.<br>13. Food Assistance/ OR ("Supplemental Nutrition Assistance Program" OR SNAP OR "emergency supplement*" OR "Meals on Wheels" OR "Meal Replacement Benefits" OR "Nutrition Assistance" OR "Nutritional Assistance" OR "Women, Infants, and Children" OR WIC).ti,ab,kf.<br>14. "Economic Impact Payment*" OR "Stimulus Check*" OR "Inflation Relief Check*" OR "Child Tax Credit" OR "American Rescue Plan"<br>15. Social Security/ OR ("Supplemental Security Income" OR social security OR disability insurance).ti,ab,kf.		

© 2024 Zhang et al. *JAMA Health Forum.*

	16. Insurance, Pharmaceutical Services/ OR State Pharmaceutical Assistance Programs.ti,ab,kf.<br>17. Exp Health policy/ec OR (Economic ADJ5 polic*).ti,ab,kf.<br>18. OR/2-17<br>19. 1 AND 18<br>20. Limit 19 to (English language and yr="2000-current")		
**Embase (OVID) 1974-**	1. Exp Hypertension/ OR (hypertension OR hypertensive OR blood pressure).ti,ab,kf.<br>2. exp "Salary and Fringe Benefit"/ OR (minimum wage* OR hourly wage* OR hourly salary).ti,ab,kf.<br>3. (Insurance/ AND (Unemployment/ OR (unemploy* OR ((job OR employ*) ADJ2 loss) OR employment status).ti,ab,kf.)) OR (unemployment insurance OR unemployment benefit* OR unemployment coverage).ti,ab,kf.<br>4. (Earned Income Tax Credit OR EITC).ti,ab,kf.<br>5. ("Temporary Assistance for Needy Families" OR TANF OR "Aid to Families with Dependent Children" OR AFDC).ti,ab,kf.<br>6. Medical Leave/ OR (Paid leave OR Sick leave).ti,ab,kf.<br>7. Family Leave/ OR ("Family and Medical Leave Act" OR FMLA OR family leave OR medical leave).ti,ab,kf.<br>8. (ACA OR Affordable Care Act OR Obamacare).ti,ab,kf.<br>9. Exp Medicaid/ OR Medicaid Expansion.ti,ab,kf.<br>10. Exp Medicare/ OR ("Part D" OR (Medicare AND (drug coverage OR drug insurance OR drug benefit* OR (prescription* ADJ2 cost*))) OR (cap ADJ5 cost*) OR "Inflation Reduction Act" OR (medicare ADJ5 (savings OR beneficiary OR fee-for-service*)) OR "Qualifying Individual Program" OR "Qualified Disabled Working Individual Program").ti,ab,kf.<br>11. ((telehealth OR telemedicine OR tele-health OR tele-medicine OR ehealth OR mhealth OR mobile health OR audio only service*) ADJ5 (parity OR polic* OR law* OR regulation* OR	11/01/2022	717<br><br>- duplicates<br><br>=543 unique items

© 2024 Zhang et al. *JAMA Health Forum.*

	payment* OR payer OR all-payer OR reimburse* OR waiver*)).ti,ab,kf.<br>12. (Housing/ AND (Public Policy/ OR health care policy/)) OR (Eviction Moratori* OR Eviction Protection* OR Housing Polic* OR Mortgage Relief OR Foreclosure Relief OR ((Home* OR housing) ADJ2 Afford*)).ti,ab,kf.<br>13. Food Assistance/ OR ("Supplemental Nutrition Assistance Program" OR SNAP OR "emergency supplement*" OR "Meals on Wheels" OR "Meal Replacement Benefits" OR "Nutrition Assistance" OR "Nutritional Assistance" OR "Women, Infants, and Children" OR WIC).ti,ab,kf.<br>14. "Economic Impact Payment*" OR "Stimulus Check*" OR "Inflation Relief Check*" OR "Child Tax Credit" OR "American Rescue Plan"<br>15. Social Security/ OR ("Supplemental Security Income" OR social security OR disability insurance).ti,ab,kf.<br>16. State Pharmaceutical Assistance Programs.ti,ab,kf.<br>17. (Economic* ADJ5 polic*).ti,ab,kf,hw.<br>18. OR/2-17<br>19. 1 AND 18<br>20. Limit 19 to (English language and yr="2000-current")<br>21. limit 20 to conference abstract status<br>22. 20 not 21<br>23. limit 22 to "remove medline records"		
**PsycInfo (OVID)**	1. (hypertension OR hypertensive OR blood pressure).ti,ab,sh,mh,hw.<br>2. (minimum wage* OR hourly wage* OR hourly salary).ti,ab,sh,mh,hw.<br>3. (unemployment insurance OR unemployment benefit* OR unemployment coverage).ti,ab,sh,mh,hw.<br>4. (Earned Income Tax Credit OR EITC).ti,ab,sh,mh,hw.<br>5. ("Temporary Assistance for Needy Families" OR TANF OR "Aid to Families with Dependent Children" OR AFDC).ti,ab,sh,mh,hw.<br>6. (Paid leave OR Sick leave).ti,ab,sh,mh,hw.	11/01/2022	147<br><br>- duplicates<br><br>=60 unique items

© 2024 Zhang et al. *JAMA Health Forum.*

		7. ("Family and Medical Leave Act" OR FMLA OR family leave OR medical leave).ti,ab,sh,mh,hw.<br>8. (ACA OR Affordable Care Act OR Obamacare).ti,ab,sh,mh,hw.<br>9. Medicaid Expansion.ti,ab,sh,mh,hw.<br>10. ("Part D" OR (Medicare AND (drug coverage OR drug insurance OR drug benefit* OR (prescription* ADJ2 cost*))) OR (cap ADJ5 cost*) OR "Inflation Reduction Act" OR (medicare ADJ5 (savings OR beneficiary OR fee-for-service*)) OR "Qualifying Individual Program" OR "Qualified Disabled Working Individual Program").ti,ab,sh,mh,hw.<br>11. ((telehealth OR telemedicine OR tele-health OR tele-medicine OR ehealth OR mhealth OR mobile health OR audio only service*) ADJ5 (parity OR polic* OR law* OR regulation* OR payment* OR payer OR all-payer OR reimburse* OR waiver*)).ti,ab,sh,mh,hw.<br>12. (Eviction Moratori* OR Eviction Protection* OR Housing Polic* OR Mortgage Relief OR Foreclosure Relief OR ((Home* OR housing) ADJ2 Afford*)).ti,ab,sh,mh,hw.<br>13. (food assistance OR "Supplemental Nutrition Assistance Program" OR SNAP OR "emergency supplement*" OR "Meals on Wheels" OR "Meal Replacement Benefits" OR "Nutrition Assistance" OR "Nutritional Assistance" OR "Women, Infants, and Children" OR WIC).ti,ab,sh,mh,hw.<br>14. "Economic Impact Payment*" OR "Stimulus Check*" OR "Inflation Relief Check*" OR "Child Tax Credit" OR "American Rescue Plan"<br>15. ("Supplemental Security Income" OR social security OR disability insurance).ti,ab,sh,mh,hw.<br>16. State Pharmaceutical Assistance Programs.ti,ab,sh,mh,hw.<br>17. (Economic* ADJ5 polic*).ti,ab,sh,mh,hw.<br>18. OR/2-17<br>19. 1 AND 18		

© 2024 Zhang et al. *JAMA Health Forum.*

	20. Limit 19 to (English language and yr="2000-current")		
**Cochrane Library**	1. [mh "Hypertension"] OR (hypertension OR hypertensive OR "blood pressure"):ti,ab<br>2. [mh "Salaries and Fringe Benefits"] OR ("minimum wage*" OR "hourly wage*" OR "hourly salary"):ti,ab<br>3. ([mh "Insurance coverage"] AND ([mh "Unemployment"] OR (unemploy* OR ((job OR employ*) NEAR/2 loss) OR "employment status"):ti,ab)) OR ("unemployment insurance" OR "unemployment benefit*" OR "unemployment coverage"):ti,ab<br>4. ("Earned Income Tax Credit" OR EITC):ti,ab<br>5. [mh "Public Assistance"] OR [mh "Aid to Families with Dependent Children"] OR ("Temporary Assistance for Needy Families" OR TANF OR "Aid to Families with Dependent Children" OR AFDC):ti,ab<br>6. [mh "Sick Leave"] OR ("Paid leave" OR "Sick leave"):ti,ab<br>7. (FMLA OR "family leave" OR "medical leave"):ti,ab<br>8. [mh "Patient Protection and Affordable Care Act"] OR (ACA OR "Affordable Care Act" OR Obamacare):ti,ab<br>9. [mh "Medicaid"] OR "Medicaid Expansion":ti,ab<br>10. [mh "Medicare"] OR [mh "Medicare Part D"] OR ("Part D" OR (Medicare AND ("drug coverage" OR "drug insurance" OR "drug benefit*" OR (prescription* NEAR/2 cost*))) OR (cap NEAR/5 cost*) OR "Inflation Reduction Act" OR (medicare NEAR/5 (savings OR beneficiary OR fee-for-service*)) OR "Qualifying Individual Program" OR "Qualified Disabled Working Individual Program"):ti,ab<br>11. ((telehealth OR telemedicine OR tele-health OR tele-medicine OR ehealth OR mhealth OR "mobile health" OR "audio only service*") NEAR/5 (parity OR polic* OR law* OR	11/01/2022	251<br><br>- duplicates<br><br>=155 unique items

© 2024 Zhang et al. *JAMA Health Forum.*

	regulation* OR payment* OR payer OR all-payer OR reimburse* OR waiver*)):ti,ab<br>12. ([mh "Housing"] AND [mh "Public Policy"]) OR ("Eviction Moratori*" OR "Eviction Protection*" OR "Housing Polic*" OR "Mortgage Relief" OR "Foreclosure Relief" OR ((Home* OR housing) NEAR/2 Afford*)):ti,ab<br>13. [mh "Food Assistance"] OR ("food assistance" OR SNAP OR "emergency supplement*" OR "Meals on Wheels" OR "Meal Replacement Benefits" OR "Nutrition Assistance" OR "Nutritional Assistance" OR "Women, Infants, and Children" OR WIC):ti,ab<br>14. ("Economic Impact Payment*" OR "Stimulus Check*" OR "Inflation Relief Check*" OR "Child Tax Credit" OR "American Rescue Plan"):ti,ab<br>15. [mh "Social Security"] OR ("Supplemental Security Income" OR "social security" OR "disability insurance"):ti,ab<br>16. [mh "Insurance, Pharmaceutical Services"] OR "State Pharmaceutical Assistance Programs":ti,ab<br>17. (Economic* NEAR/5 polic*):ti,ab<br>18. OR/#2-#17<br>19. #1 AND #18<br><br>Limit 2000 -		
**CINAHL (EBSCOHost)**	S1 (MH "Hypertension") OR (hypertension OR hypertensive OR "blood pressure")<br><br>S2 (MH "Salaries and Fringe Benefits") OR ("minimum wage*" OR "hourly wage*" OR "hourly salary") OR (MH "Insurance, Unemployment") OR ((MH "Insurance coverage") AND ((MH "Unemployment") OR (unemploy* OR ((job OR employ*) N2 loss) OR "employment status"))) OR ("unemployment insurance" OR "unemployment benefit*" OR "unemployment coverage") OR ("Earned Income Tax Credit" OR EITC) OR (MH "Public Assistance") OR (MH "Aid to Families with Dependent Children") OR ("Temporary Assistance for Needy Families" OR TANF OR "Aid to Families with Dependent Children" OR AFDC) OR (MH "Sick Leave") OR ("Paid leave" OR "Sick leave") OR (MH "Family and Medical Leave") OR (FMLA OR "family leave" OR "medical leave") OR (MH "Patient Protection	11/01/2022	506<br><br>- duplicates<br><br>=239 unique items

© 2024 Zhang et al. *JAMA Health Forum.*

	and Affordable Care Act") OR (ACA OR "Affordable Care Act" OR Obamacare) OR (MH "United States Centers for Medicare and Medicaid Services") OR (MH "Medicaid") OR "Medicaid Expansion" OR (MH "Medicare") OR ("Part D" OR (Medicare AND ("drug coverage" OR "drug insurance" OR "drug benefit*" OR (prescription* N2 cost*))) OR (cap N5 cost*) OR "Inflation Reduction Act" OR (medicare N5 (savings OR beneficiary OR fee-for-service*)) OR "Qualifying Individual Program" OR "Qualified Disabled Working Individual Program") OR ((telehealth OR telemedicine OR tele-health OR tele-medicine OR ehealth OR mhealth OR "mobile health" OR "audio only service*") N5 (parity OR polic* OR law* OR regulation* OR payment* OR payer OR all-payer OR reimburse* OR waiver*)) OR ((MH "Housing") AND (MH "Public Policy")) OR ("Eviction Moratori*" OR "Eviction Protection*" OR "Housing Polic*" OR "Mortgage Relief" OR "Foreclosure Relief" OR ((Home* OR housing) N2 Afford*)) OR (MH "Food Assistance") OR ("food assistance" OR SNAP OR "emergency supplement*" OR "Meals on Wheels" OR "Meal Replacement Benefits" OR "Nutrition Assistance" OR "Nutritional Assistance" OR "Women, Infants, and Children" OR WIC) OR ("Economic Impact Payment*" OR "Stimulus Check*" OR "Inflation Relief Check*" OR "Child Tax Credit" OR "American Rescue Plan") OR ("Supplemental Security Income" OR "social security" OR "disability insurance") OR (MH "Insurance, Pharmaceutical Services") OR "State Pharmaceutical Assistance Programs" OR ((MH "Health policy") AND economic*) OR (Economic* N5 polic*)<br><br>S1 AND S2<br><br>**Limiters** - Published Date: 20000101-20221231; English Language; Exclude MEDLINE records		

© 2024 Zhang et al. *JAMA Health Forum.*

**EconLit (EBSCOHost)**	S1 (hypertension OR hypertensive OR "blood pressure")<br><br>S2 ("minimum wage*" OR "hourly wage*" OR "hourly salary") OR (insurance AND (unemploy* OR ((job OR employ*) NEAR/2 loss) OR "employment status"))) OR ("unemployment insurance" OR "unemployment benefit*" OR "unemployment coverage") OR ("Earned Income Tax Credit" OR EITC) OR ("Temporary Assistance for Needy Families" OR TANF OR "Aid to Families with Dependent Children" OR AFDC) OR ("Paid leave" OR "Sick leave") OR (FMLA OR "family leave" OR "medical leave") OR (ACA OR "Affordable Care Act" OR Obamacare) OR "Medicaid Expansion" OR ("Part D" OR (Medicare AND ("drug coverage" OR "drug insurance" OR "drug benefit*" OR (prescription* N2 cost*))) OR (cap N5 cost*) OR "Inflation Reduction Act" OR (medicare N5 (savings OR beneficiary OR fee-for-service*)) OR "Qualifying Individual Program" OR "Qualified Disabled Working Individual Program") OR ((telehealth OR telemedicine OR tele-health OR tele-medicine OR ehealth OR mhealth OR "mobile health" OR "audio only service*") N5 (parity OR polic* OR law* OR regulation* OR payment* OR payer OR all-payer OR reimburse* OR waiver*)) OR (Housing AND "Public Policy") OR ("Eviction Moratori*" OR "Eviction Protection*" OR "Housing Polic*" OR "Mortgage Relief" OR "Foreclosure Relief" OR ((Home* OR housing) N2 Afford*)) OR ("food assistance" OR SNAP OR "emergency supplement*" OR "Meals on Wheels" OR "Meal Replacement Benefits" OR "Nutrition Assistance" OR "Nutritional Assistance" OR "Women, Infants, and Children" OR WIC) OR ("Economic Impact Payment*" OR "Stimulus Check*" OR "Inflation Relief Check*" OR "Child Tax Credit" OR "American Rescue Plan") OR ("Supplemental Security Income" OR "social security" OR "disability insurance") OR "State Pharmaceutical Assistance Programs" OR (Economic* N5 polic*)<br><br>**Limiters** - Published Date: 20000101-20221231; English Language;	11/01/2022	52<br><br>- duplicates<br><br>=43 unique items

© 2024 Zhang et al. *JAMA Health Forum.*

**Sociological Abstracts (ProQuest)**	TI,AB,SU(hypertension OR hypertensive OR "blood pressure")<br>AND<br>("minimum wage*" OR "hourly wage*" OR "hourly salary" OR (insurance AND (unemploy* OR (job loss OR (employ* NEAR/2 loss) OR "employment status"))) OR "unemployment insurance" OR "unemployment benefit*" OR "unemployment coverage" OR "Earned Income Tax Credit" OR EITC OR "Temporary Assistance for Needy Families" OR TANF OR "Aid to Families with Dependent Children" OR AFDC OR "Paid leave" OR "Sick leave" OR FMLA OR "family leave" OR "medical leave" OR ACA OR "Affordable Care Act" OR Obamacare OR "Medicaid Expansion" OR "Part D" OR (Medicare AND ("drug coverage" OR "drug insurance" OR "drug benefit*" OR (prescription* NEAR/2 cost*))) OR (cap NEAR/5 cost*) OR "Inflation Reduction Act" OR (medicare NEAR/5 (savings OR beneficiary OR fee-for-service*)) OR "Qualifying Individual Program" OR "Qualified Disabled Working Individual Program" OR ((telehealth OR telemedicine OR tele-health OR tele-medicine OR ehealth OR mhealth OR "mobile health" OR "audio only service*") NEAR/5 (parity OR polic* OR law* OR regulation* OR payment* OR payer OR all-payer OR reimburse* OR waiver*)) OR (Housing AND "Public Policy") OR "Eviction Moratori*" OR "Eviction Protection*" OR "Housing Polic*" OR "Mortgage Relief" OR "Foreclosure Relief" OR ((Home* OR housing) NEAR/2 Afford*) OR "food assistance" OR SNAP OR "emergency supplement*" OR "Meals on Wheels" OR "Meal Replacement Benefits" OR "Nutrition Assistance" OR "Nutritional Assistance" OR "Women, Infants, and Children" OR WIC OR "Economic Impact Payment*" OR "Stimulus Check*" OR "Inflation Relief Check*" OR "Child Tax Credit" OR "American Rescue Plan" OR "Supplemental Security Income" OR "social security" OR "disability insurance" OR "State Pharmaceutical Assistance Programs" OR (Economic* NEAR/5 polic*)<br>Limit English; 2000 - current	11/01/2022	208<br><br>- duplicates<br><br>=195 unique items
**Scopus**	TITLE-ABS-KEY(hypertension OR hypertensive OR "blood pressure") AND TITLE-ABS-KEY("minimum wage*" OR "hourly wage*" OR "hourly salary" OR (insurance AND (unemploy* OR (job loss OR (employ* W/2 loss) OR "employment status"))) OR	11/01/2022	456<br><br>- duplicates

© 2024 Zhang et al. *JAMA Health Forum.*

	"unemployment insurance" OR "unemployment benefit*" OR "unemployment coverage" OR "Earned Income Tax Credit" OR EITC OR "Temporary Assistance for Needy Families" OR TANF OR "Aid to Families with Dependent Children" OR AFDC OR "Paid leave" OR "Sick leave" OR FMLA OR "family leave" OR "medical leave" OR ACA OR "Affordable Care Act" OR Obamacare OR "Medicaid Expansion" OR "Part D" OR (Medicare AND ("drug coverage" OR "drug insurance" OR "drug benefit*" OR (prescription* W/2 cost*))) OR (cap W/5 cost*) OR "Inflation Reduction Act" OR (medicare W/5 (savings OR beneficiary OR fee-for-service*)) OR "Qualifying Individual Program" OR "Qualified Disabled Working Individual Program" OR ((telehealth OR telemedicine OR tele-health OR tele-medicine OR ehealth OR mhealth OR "mobile health" OR "audio only service*") W/5 (parity OR polic* OR law* OR regulation* OR payment* OR payer OR all-payer OR reimburse* OR waiver*)) OR (Housing AND "Public Policy") OR "Eviction Moratori*" OR "Eviction Protection*" OR "Housing Polic*" OR "Mortgage Relief" OR "Foreclosure Relief" OR ((Home* OR housing) W/2 Afford*) OR "food assistance" OR SNAP OR "emergency supplement*" OR "Meals on Wheels" OR "Meal Replacement Benefits" OR "Nutrition Assistance" OR "Nutritional Assistance" OR "Women, Infants, and Children" OR WIC OR "Economic Impact Payment*" OR "Stimulus Check*" OR "Inflation Relief Check*" OR "Child Tax Credit" OR "American Rescue Plan" OR "Supplemental Security Income" OR "social security" OR "disability insurance" OR "State Pharmaceutical Assistance Programs" OR (Economic* W/5 polic*)) AND NOT INDEX(medline)<br>Limit English; 2000 - current		=195 unique items

Notes: Duplicates were identified using the Endnote automated "find duplicates" function with preference set to match on title, author, and year, and removed from your Endnote library. There will likely be additional duplicates found that Endnote was unable to detect.

eTable 1 (Cont.) Second Round of Literature Search Results (11/1/2022 – 6/14/2023)

© 2024 Zhang et al. *JAMA Health Forum.*

Search Strategy:

Database	Strategy	Records 11/01/2022	Records 06/14/2023
**Medline (OVID) 1946-**	21. Exp Hypertension/ OR (hypertension OR hypertensive OR blood pressure).ti,ab,kf.<br>22. exp "Salaries and Fringe Benefits"/ OR (minimum wage* OR hourly wage* OR hourly salary).ti,ab,kf.<br>23. (Insurance coverage/ AND (Unemployment/ OR (unemploy* OR ((job OR employ*) ADJ2 loss) OR employment status).ti,ab,kf.)) OR (unemployment insurance OR unemployment benefit* OR unemployment coverage).ti,ab,kf.<br>24. (Earned Income Tax Credit OR EITC).ti,ab,kf.<br>25. Exp Public Assistance/ OR Aid to Families with Dependent Children/ OR ("Temporary Assistance for Needy Families" OR TANF OR "Aid to Families with Dependent Children" OR AFDC).ti,ab,kf.<br>26. Sick Leave/ OR (Paid leave OR Sick leave).ti,ab,kf.<br>27. Family Leave/ OR ("Family and Medical Leave Act" OR FMLA OR family leave OR medical leave).ti,ab,kf.<br>28. "Patient Protection and Affordable Care Act"/ OR (ACA OR Affordable Care Act OR Obamacare).ti,ab,kf.<br>29. "Centers for Medicare and Medicaid Services, U.S."/ OR Medicaid/ OR Dual MEDICAID MEDICARE Eligibility/ OR Medicaid Expansion.ti,ab,kf.<br>30. Exp Medicare/ OR Medicare Part D/ OR ("Part D" OR (Medicare AND (drug coverage OR drug insurance OR drug benefit* OR (prescription* ADJ2 cost*))) OR (cap ADJ5 cost*) OR "Inflation Reduction Act" OR (medicare ADJ5 (savings OR beneficiary OR fee-for-service*)) OR "Qualifying Individual Program" OR "Qualified Disabled Working Individual Program").ti,ab,kf.<br>31. "All-Payer Telemedicine Parity" OR "Medicaid reimbursement for audio-only services" OR "Medicare Expansion of Telehealth with 1135 Waiver" OR ((telehealth OR telemedicine OR tele-health OR tele-medicine OR ehealth OR mhealth	2127	121

© 2024 Zhang et al. *JAMA Health Forum.*

	OR mobile health OR audio only service*) ADJ5 (parity OR polic* OR law* OR regulation* OR payment* OR payer OR all-payer OR reimburse* OR waiver*)).ti,ab,kf,hw.<br>32. (Housing/ AND Public Policy/) OR (Eviction Moratori* OR Eviction Protection* OR Housing Polic* OR Mortgage Relief OR Foreclosure Relief OR ((Home* OR housing) ADJ2 Afford*)).ti,ab,kf.<br>33. Food Assistance/ OR ("Supplemental Nutrition Assistance Program" OR SNAP OR "emergency supplement*" OR "Meals on Wheels" OR "Meal Replacement Benefits" OR "Nutrition Assistance" OR "Nutritional Assistance" OR "Women, Infants, and Children" OR WIC).ti,ab,kf.<br>34. "Economic Impact Payment*" OR "Stimulus Check*" OR "Inflation Relief Check*" OR "Child Tax Credit" OR "American Rescue Plan"<br>35. Social Security/ OR ("Supplemental Security Income" OR social security OR disability insurance).ti,ab,kf.<br>36. Insurance, Pharmaceutical Services/ OR State Pharmaceutical Assistance Programs.ti,ab,kf.<br>37. Exp Health policy/ec OR (Economic ADJ5 polic*).ti,ab,kf.<br>38. OR/2-17<br>39. 1 AND 18<br>40. Limit 19 to (English language and yr="2000-current")		
**Embase (OVID) 1974-**	24. Exp Hypertension/ OR (hypertension OR hypertensive OR blood pressure).ti,ab,kf.<br>25. exp "Salary and Fringe Benefit"/ OR (minimum wage* OR hourly wage* OR hourly salary).ti,ab,kf.<br>26. (Insurance/ AND (Unemployment/ OR (unemploy* OR ((job OR employ*) ADJ2 loss) OR employment status).ti,ab,kf.)) OR (unemployment insurance OR unemployment benefit* OR unemployment coverage).ti,ab,kf.<br>27. (Earned Income Tax Credit OR EITC).ti,ab,kf.	717<br><br>- duplicates<br><br>=543 unique items	96<br><br>- duplicates<br><br>=87 unique items

© 2024 Zhang et al. *JAMA Health Forum.*

	28. ("Temporary Assistance for Needy Families" OR TANF OR "Aid to Families with Dependent Children" OR AFDC).ti,ab,kf.<br>29. Medical Leave/ OR (Paid leave OR Sick leave).ti,ab,kf.<br>30. Family Leave/ OR ("Family and Medical Leave Act" OR FMLA OR family leave OR medical leave).ti,ab,kf.<br>31. (ACA OR Affordable Care Act OR Obamacare).ti,ab,kf.<br>32. Exp Medicaid/ OR Medicaid Expansion.ti,ab,kf.<br>33. Exp Medicare/ OR ("Part D" OR (Medicare AND (drug coverage OR drug insurance OR drug benefit* OR (prescription* ADJ2 cost*))) OR (cap ADJ5 cost*) OR "Inflation Reduction Act" OR (medicare ADJ5 (savings OR beneficiary OR fee-for-service*)) OR "Qualifying Individual Program" OR "Qualified Disabled Working Individual Program").ti,ab,kf.<br>34. ((telehealth OR telemedicine OR tele-health OR tele-medicine OR ehealth OR mhealth OR mobile health OR audio only service*) ADJ5 (parity OR polic* OR law* OR regulation* OR payment* OR payer OR all-payer OR reimburse* OR waiver*)).ti,ab,kf.<br>35. (Housing/ AND (Public Policy/ OR health care policy/)) OR (Eviction Moratori* OR Eviction Protection* OR Housing Polic* OR Mortgage Relief OR Foreclosure Relief OR ((Home* OR housing) ADJ2 Afford*)).ti,ab,kf.<br>36. Food Assistance/ OR ("Supplemental Nutrition Assistance Program" OR SNAP OR "emergency supplement*" OR "Meals on Wheels" OR "Meal Replacement Benefits" OR "Nutrition Assistance" OR "Nutritional Assistance" OR "Women, Infants, and Children" OR WIC).ti,ab,kf.<br>37. "Economic Impact Payment*" OR "Stimulus Check*" OR "Inflation Relief Check*" OR "Child Tax Credit" OR "American Rescue Plan"<br>38. Social Security/ OR ("Supplemental Security Income" OR social security OR disability insurance).ti,ab,kf.		

© 2024 Zhang et al. *JAMA Health Forum.*

	39. State Pharmaceutical Assistance Programs.ti,ab,kf.<br>40. (Economic* ADJ5 polic*).ti,ab,kf,hw.<br>41. OR/2-17<br>42. 1 AND 18<br>43. Limit 19 to (English language and yr="2000-current")<br>44. limit 20 to conference abstract status<br>45. 20 not 21<br>46. limit 22 to "remove medline records"		
**PsycInfo (OVID)**	21. (hypertension OR hypertensive OR blood pressure).ti,ab,sh,mh,hw.<br>22. (minimum wage* OR hourly wage* OR hourly salary).ti,ab,sh,mh,hw.<br>23. (unemployment insurance OR unemployment benefit* OR unemployment coverage).ti,ab,sh,mh,hw.<br>24. (Earned Income Tax Credit OR EITC).ti,ab,sh,mh,hw.<br>25. ("Temporary Assistance for Needy Families" OR TANF OR "Aid to Families with Dependent Children" OR AFDC).ti,ab,sh,mh,hw.<br>26. (Paid leave OR Sick leave).ti,ab,sh,mh,hw.<br>27. ("Family and Medical Leave Act" OR FMLA OR family leave OR medical leave).ti,ab,sh,mh,hw.<br>28. (ACA OR Affordable Care Act OR Obamacare).ti,ab,sh,mh,hw.<br>29. Medicaid Expansion.ti,ab,sh,mh,hw.<br>30. ("Part D" OR (Medicare AND (drug coverage OR drug insurance OR drug benefit* OR (prescription* ADJ2 cost*))) OR (cap ADJ5 cost*) OR "Inflation Reduction Act" OR (medicare ADJ5 (savings OR beneficiary OR fee-for-service*)) OR "Qualifying Individual Program" OR "Qualified Disabled Working Individual Program").ti,ab,sh,mh,hw.<br>31. ((telehealth OR telemedicine OR tele-health OR tele-medicine OR ehealth OR mhealth OR mobile health OR audio only service*) ADJ5 (parity OR polic* OR law* OR regulation* OR payment* OR	147<br><br>- duplicates<br><br>=60 unique items	3<br><br>- duplicates<br><br>=2 unique items

© 2024 Zhang et al. *JAMA Health Forum.*

	payer OR all-payer OR reimburse* OR waiver*)).ti,ab,sh,mh,hw.<br>32. (Eviction Moratori* OR Eviction Protection* OR Housing Polic* OR Mortgage Relief OR Foreclosure Relief OR ((Home* OR housing) ADJ2 Afford*)).ti,ab,sh,mh,hw.<br>33. (food assistance OR "Supplemental Nutrition Assistance Program" OR SNAP OR "emergency supplement*" OR "Meals on Wheels" OR "Meal Replacement Benefits" OR "Nutrition Assistance" OR "Nutritional Assistance" OR "Women, Infants, and Children" OR WIC).ti,ab,sh,mh,hw.<br>34. "Economic Impact Payment*" OR "Stimulus Check*" OR "Inflation Relief Check*" OR "Child Tax Credit" OR "American Rescue Plan"<br>35. ("Supplemental Security Income" OR social security OR disability insurance).ti,ab,sh,mh,hw.<br>36. State Pharmaceutical Assistance Programs.ti,ab,sh,mh,hw.<br>37. (Economic* ADJ5 polic*).ti,ab,sh,mh,hw.<br>38. OR/2-17<br>39. 1 AND 18<br>40. Limit 19 to (English language and yr="2000-current")		
**Cochrane Library**	20. [mh "Hypertension"] OR (hypertension OR hypertensive OR "blood pressure"):ti,ab<br>21. [mh "Salaries and Fringe Benefits"] OR ("minimum wage*" OR "hourly wage*" OR "hourly salary"):ti,ab<br>22. ([mh "Insurance coverage"] AND ([mh "Unemployment"] OR (unemploy* OR ((job OR employ*) NEAR/2 loss) OR "employment status"):ti,ab)) OR ("unemployment insurance" OR "unemployment benefit*" OR "unemployment coverage"):ti,ab<br>23. ("Earned Income Tax Credit" OR EITC):ti,ab<br>24. [mh "Public Assistance"] OR [mh "Aid to Families with Dependent Children"] OR ("Temporary Assistance for Needy Families" OR TANF OR "Aid to Families with Dependent Children" OR AFDC):ti,ab	251<br><br>- duplicates<br><br>=155 unique items	11<br><br>- duplicates<br><br>=8 unique items

© 2024 Zhang et al. *JAMA Health Forum.*

	25. [mh "Sick Leave"] OR ("Paid leave" OR "Sick leave"):ti,ab<br>26. (FMLA OR "family leave" OR "medical leave"):ti,ab<br>27. [mh "Patient Protection and Affordable Care Act"] OR (ACA OR "Affordable Care Act" OR Obamacare):ti,ab<br>28. [mh "Medicaid"] OR "Medicaid Expansion":ti,ab<br>29. [mh "Medicare"] OR [mh "Medicare Part D"] OR ("Part D" OR (Medicare AND ("drug coverage" OR "drug insurance" OR "drug benefit*" OR (prescription* NEAR/2 cost*))) OR (cap NEAR/5 cost*) OR "Inflation Reduction Act" OR (medicare NEAR/5 (savings OR beneficiary OR fee-for-service*)) OR "Qualifying Individual Program" OR "Qualified Disabled Working Individual Program"):ti,ab<br>30. ((telehealth OR telemedicine OR tele-health OR tele-medicine OR ehealth OR mhealth OR "mobile health" OR "audio only service*") NEAR/5 (parity OR polic* OR law* OR regulation* OR payment* OR payer OR all-payer OR reimburse* OR waiver*)):ti,ab<br>31. ([mh "Housing"] AND [mh "Public Policy"]) OR ("Eviction Moratori*" OR "Eviction Protection*" OR "Housing Polic*" OR "Mortgage Relief" OR "Foreclosure Relief" OR ((Home* OR housing) NEAR/2 Afford*)):ti,ab<br>32. [mh "Food Assistance"] OR ("food assistance" OR SNAP OR "emergency supplement*" OR "Meals on Wheels" OR "Meal Replacement Benefits" OR "Nutrition Assistance" OR "Nutritional Assistance" OR "Women, Infants, and Children" OR WIC):ti,ab<br>33. ("Economic Impact Payment*" OR "Stimulus Check*" OR "Inflation Relief Check*" OR "Child Tax Credit" OR "American Rescue Plan"):ti,ab<br>34. [mh "Social Security"] OR ("Supplemental Security Income" OR "social security" OR "disability insurance"):ti,ab<br>35. [mh "Insurance, Pharmaceutical Services"] OR "State Pharmaceutical Assistance Programs":ti,ab		

© 2024 Zhang et al. *JAMA Health Forum.*

	36. (Economic* NEAR/5 polic*):ti,ab<br>37. OR/#2-#17<br>38. #1 AND #18<br><br>Limit 2000 -		
**CINAHL (EBSCOHost)**	S1 (MH "Hypertension") OR (hypertension OR hypertensive OR "blood pressure")<br><br>S2 (MH "Salaries and Fringe Benefits") OR ("minimum wage*" OR "hourly wage*" OR "hourly salary") OR (MH "Insurance, Unemployment") OR ((MH "Insurance coverage") AND ((MH "Unemployment") OR (unemploy* OR ((job OR employ*) N2 loss) OR "employment status"))) OR ("unemployment insurance" OR "unemployment benefit*" OR "unemployment coverage") OR ("Earned Income Tax Credit" OR EITC) OR (MH "Public Assistance") OR (MH "Aid to Families with Dependent Children") OR ("Temporary Assistance for Needy Families" OR TANF OR "Aid to Families with Dependent Children" OR AFDC) OR (MH "Sick Leave") OR ("Paid leave" OR "Sick leave") OR (MH "Family and Medical Leave") OR (FMLA OR "family leave" OR "medical leave") OR (MH "Patient Protection and Affordable Care Act") OR (ACA OR "Affordable Care Act" OR Obamacare) OR (MH "United States Centers for Medicare and Medicaid Services") OR (MH "Medicaid") OR "Medicaid Expansion" OR (MH "Medicare") OR ("Part D" OR (Medicare AND ("drug coverage" OR "drug insurance" OR "drug benefit*" OR (prescription* N2 cost*))) OR (cap N5 cost*) OR "Inflation Reduction Act" OR (medicare N5 (savings OR beneficiary OR fee-for-service*)) OR "Qualifying Individual Program" OR "Qualified Disabled Working Individual Program") OR ((telehealth OR telemedicine OR tele-health OR tele-medicine OR ehealth OR mhealth OR "mobile health" OR "audio only service*") N5 (parity OR polic* OR law* OR regulation* OR payment* OR payer OR all-payer OR reimburse* OR waiver*)) OR ((MH	506<br><br>-<br>duplicates<br><br>=239<br>unique<br>items	25<br><br>-<br>duplicates<br><br>=9<br>unique<br>items

© 2024 Zhang et al. *JAMA Health Forum.*

	"Housing") AND (MH "Public Policy")) OR ("Eviction Moratori*" OR "Eviction Protection*" OR "Housing Polic*" OR "Mortgage Relief" OR "Foreclosure Relief" OR ((Home* OR housing) N2 Afford*)) OR (MH "Food Assistance") OR ("food assistance" OR SNAP OR "emergency supplement*" OR "Meals on Wheels" OR "Meal Replacement Benefits" OR "Nutrition Assistance" OR "Nutritional Assistance" OR "Women, Infants, and Children" OR WIC) OR ("Economic Impact Payment*" OR "Stimulus Check*" OR "Inflation Relief Check*" OR "Child Tax Credit" OR "American Rescue Plan") OR ("Supplemental Security Income" OR "social security" OR "disability insurance") OR (MH "Insurance, Pharmaceutical Services") OR "State Pharmaceutical Assistance Programs" OR ((MH "Health policy") AND economic*) OR (Economic* N5 polic*)<br><br>S1 AND S2<br><br>**Limiters** - Published Date: 20000101-20221231; English Language; Exclude MEDLINE records<br>**Limiters** - Published Date: 20221101-20230631; English Language; Exclude MEDLINE records		
**EconLit (EBSCOHost)**	S1 (hypertension OR hypertensive OR "blood pressure")<br><br>S2 ("minimum wage*" OR "hourly wage*" OR "hourly salary") OR (insurance AND (unemploy* OR ((job OR employ*) NEAR/2 loss) OR "employment status"))) OR ("unemployment insurance" OR "unemployment benefit*" OR "unemployment coverage") OR ("Earned Income Tax Credit" OR EITC) OR ("Temporary Assistance for Needy Families" OR TANF OR "Aid to Families with Dependent Children" OR AFDC) OR ("Paid leave" OR "Sick leave") OR (FMLA OR "family leave" OR "medical leave") OR (ACA OR "Affordable Care Act" OR Obamacare) OR "Medicaid Expansion" OR ("Part D" OR (Medicare AND ("drug coverage" OR "drug insurance" OR "drug benefit*" OR (prescription* N2 cost*))) OR (cap N5 cost*) OR "Inflation Reduction Act" OR (medicare N5 (savings OR	52<br><br>- duplicates<br><br>=43 unique items	3<br><br>- duplicates<br><br>=3 unique items

© 2024 Zhang et al. *JAMA Health Forum.*

	beneficiary OR fee-for-service*)) OR "Qualifying Individual Program" OR "Qualified Disabled Working Individual Program") OR ((telehealth OR telemedicine OR tele-health OR tele-medicine OR ehealth OR mhealth OR "mobile health" OR "audio only service*") N5 (parity OR polic* OR law* OR regulation* OR payment* OR payer OR all-payer OR reimburse* OR waiver*)) OR (Housing AND "Public Policy") OR ("Eviction Moratori*" OR "Eviction Protection*" OR "Housing Polic*" OR "Mortgage Relief" OR "Foreclosure Relief" OR ((Home* OR housing) N2 Afford*)) OR ("food assistance" OR SNAP OR "emergency supplement*" OR "Meals on Wheels" OR "Meal Replacement Benefits" OR "Nutrition Assistance" OR "Nutritional Assistance" OR "Women, Infants, and Children" OR WIC) OR ("Economic Impact Payment*" OR "Stimulus Check*" OR "Inflation Relief Check*" OR "Child Tax Credit" OR "American Rescue Plan") OR ("Supplemental Security Income" OR "social security" OR "disability insurance") OR "State Pharmaceutical Assistance Programs" OR (Economic* N5 polic*)<br>**Limiters** - Published Date: 20000101-20221231; English Language;		
**Sociological Abstracts (ProQuest)**	TI,AB,SU(hypertension OR hypertensive OR "blood pressure")<br>AND<br>("minimum wage*" OR "hourly wage*" OR "hourly salary" OR (insurance AND (unemploy* OR (job loss OR (employ* NEAR/2 loss) OR "employment status"))) OR "unemployment insurance" OR "unemployment benefit*" OR "unemployment coverage" OR "Earned Income Tax Credit" OR EITC OR "Temporary Assistance for Needy Families" OR TANF OR "Aid to Families with Dependent Children" OR AFDC OR "Paid leave" OR "Sick leave" OR FMLA OR "family leave" OR "medical leave" OR ACA OR "Affordable Care Act" OR Obamacare OR "Medicaid Expansion" OR "Part D" OR (Medicare AND ("drug coverage" OR "drug insurance" OR "drug benefit*" OR (prescription* NEAR/2 cost*))) OR (cap NEAR/5 cost*) OR "Inflation Reduction Act" OR (medicare NEAR/5 (savings OR beneficiary OR fee-for-service*)) OR "Qualifying Individual Program" OR	208<br>- duplicates<br>=195 unique items	11<br>- duplicates<br>=11 unique items

© 2024 Zhang et al. *JAMA Health Forum.*

	"Qualified Disabled Working Individual Program" OR ((telehealth OR telemedicine OR tele-health OR tele-medicine OR ehealth OR mhealth OR "mobile health" OR "audio only service*") NEAR/5 (parity OR polic* OR law* OR regulation* OR payment* OR payer OR all-payer OR reimburse* OR waiver*)) OR (Housing AND "Public Policy") OR "Eviction Moratori*" OR "Eviction Protection*" OR "Housing Polic*" OR "Mortgage Relief" OR "Foreclosure Relief" OR ((Home* OR housing) NEAR/2 Afford*) OR "food assistance" OR SNAP OR "emergency supplement*" OR "Meals on Wheels" OR "Meal Replacement Benefits" OR "Nutrition Assistance" OR "Nutritional Assistance" OR "Women, Infants, and Children" OR WIC OR "Economic Impact Payment*" OR "Stimulus Check*" OR "Inflation Relief Check*" OR "Child Tax Credit" OR "American Rescue Plan" OR "Supplemental Security Income" OR "social security" OR "disability insurance" OR "State Pharmaceutical Assistance Programs" OR (Economic* NEAR/5 polic*))<br><br>Limit English; 2000 - current		
**Scopus**	TITLE-ABS-KEY(hypertension OR hypertensive OR "blood pressure") AND TITLE-ABS-KEY("minimum wage*" OR "hourly wage*" OR "hourly salary" OR (insurance AND (unemploy* OR (job loss OR (employ* W/2 loss) OR "employment status"))) OR "unemployment insurance" OR "unemployment benefit*" OR "unemployment coverage" OR "Earned Income Tax Credit" OR EITC OR "Temporary Assistance for Needy Families" OR TANF OR "Aid to Families with Dependent Children" OR AFDC OR "Paid leave" OR "Sick leave" OR FMLA OR "family leave" OR "medical leave" OR ACA OR "Affordable Care Act" OR Obamacare OR "Medicaid Expansion" OR "Part D" OR (Medicare AND ("drug coverage" OR "drug insurance" OR "drug benefit*" OR (prescription* W/2 cost*))) OR (cap W/5 cost*) OR "Inflation Reduction Act" OR (medicare W/5 (savings OR beneficiary OR fee-for-service*)) OR "Qualifying Individual Program" OR "Qualified Disabled Working Individual Program" OR ((telehealth OR telemedicine OR tele-health OR tele-medicine OR ehealth OR mhealth OR "mobile health" OR "audio only service*") W/5 (parity OR polic* OR law* OR regulation* OR payment* OR payer OR all-payer OR reimburse* OR waiver*)) OR (Housing AND "Public	456<br><br>- duplicates<br><br>=195 unique items	35<br><br>- duplicates<br><br>=16 unique items

© 2024 Zhang et al. *JAMA Health Forum.*

	Policy") OR "Eviction Moratori*" OR "Eviction Protection*" OR "Housing Polic*" OR "Mortgage Relief" OR "Foreclosure Relief" OR ((Home* OR housing) W/2 Afford*) OR "food assistance" OR SNAP OR "emergency supplement*" OR "Meals on Wheels" OR "Meal Replacement Benefits" OR "Nutrition Assistance" OR "Nutritional Assistance" OR "Women, Infants, and Children" OR WIC OR "Economic Impact Payment*" OR "Stimulus Check*" OR "Inflation Relief Check*" OR "Child Tax Credit" OR "American Rescue Plan" OR "Supplemental Security Income" OR "social security" OR "disability insurance" OR "State Pharmaceutical Assistance Programs" OR (Economic* W/5 polic*)) AND NOT INDEX(medline)<br>Limit English; 2000 - current		

Notes: Duplicates were identified using the Endnote automated "find duplicates" function with preference set to match on title, author, and year, and removed from your Endnote library. There will likely be additional duplicates found that Endnote was unable to detect.

eTable 1 (Cont.) Third Round of Literature Search Results (1/1/2000 – 11/1/2023)

**Database**	**Strategy**	**Records** 11/01/2023
**Medline (OVID) 1946-**	1. Exp Hypertension/ OR exp Antihypertensive Agents/ OR exp Angiotensin-Converting Enzyme Inhibitors/ OR (hypertension OR hypertensive OR blood pressure OR antihypertensive* OR anti-hypertensive* OR beta blocker* OR diuretic* OR alpha blocker* OR vasodilator* OR ace inhibitor*).ti,ab,kf.<br>2. exp "Salaries and Fringe Benefits"/ OR (minimum wage* OR hourly wage* OR hourly salary).ti,ab,kf.<br>3. (Insurance coverage/ AND (Unemployment/ OR (unemploy* OR ((job OR employ*) ADJ2 loss) OR employment status).ti,ab,kf.)) OR (unemployment insurance OR unemployment benefit* OR unemployment coverage).ti,ab,kf.<br>4. (Earned Income Tax Credit OR EITC).ti,ab,kf.<br>5. Exp Public Assistance/ OR Aid to Families with Dependent Children/ OR ("Temporary Assistance for Needy Families" OR TANF OR "Aid to Families with Dependent Children" OR AFDC).ti,ab,kf.<br>6. Sick Leave/ OR (Paid leave OR Sick leave).ti,ab,kf.<br>7. Family Leave/ OR ("Family and Medical Leave Act" OR FMLA OR family leave OR medical leave).ti,ab,kf.	3172

	8. "Patient Protection and Affordable Care Act"/ OR (ACA OR Affordable Care Act OR Obamacare).ti,ab,kf.<br>9. "Centers for Medicare and Medicaid Services, U.S."/ OR Medicaid/ OR Dual MEDICAID MEDICARE Eligibility/ OR Medicaid Expansion.ti,ab,kf.<br>10. Exp Medicare/ OR Medicare Part D/ OR ("Part D" OR (Medicare AND (drug coverage OR drug insurance OR drug benefit* OR (prescription* ADJ2 cost*))) OR (cap ADJ5 cost*) OR "Inflation Reduction Act" OR (medicare ADJ5 (savings OR beneficiary OR fee-for-service*)) OR "Qualifying Individual Program" OR "Qualified Disabled Working Individual Program").ti,ab,kf.<br>11. "All-Payer Telemedicine Parity" OR "Medicaid reimbursement for audio-only services" OR "Medicare Expansion of Telehealth with 1135 Waiver" OR ((telehealth OR telemedicine OR tele-health OR tele-medicine OR ehealth OR mhealth OR mobile health OR audio only service*) ADJ5 (parity OR polic* OR law* OR regulation* OR payment* OR payer OR all-payer OR reimburse* OR waiver*)).ti,ab,kf,hw.<br>12. (Housing/ AND Public Policy/) OR (Eviction Moratori* OR Eviction Protection* OR Housing Polic* OR Mortgage Relief OR Foreclosure Relief OR ((Home* OR housing) ADJ2 Afford*)).ti,ab,kf.<br>13. Food Assistance/ OR ("Supplemental Nutrition Assistance Program" OR SNAP OR "emergency supplement*" OR "Meals on Wheels" OR "Meal Replacement Benefits" OR "Nutrition Assistance" OR "Nutritional Assistance" OR "Women, Infants, and Children" OR WIC).ti,ab,kf.<br>14. "Economic Impact Payment*" OR "Stimulus Check*" OR "Inflation Relief Check*" OR "Child Tax Credit" OR "American Rescue Plan"<br>15. Social Security/ OR ("Supplemental Security Income" OR social security OR disability insurance).ti,ab,kf.<br>16. Insurance, Pharmaceutical Services/ OR State Pharmaceutical Assistance Program*.ti,ab,kf.<br>17. Exp Health policy/ec OR (Economic ADJ5 polic*).ti,ab,kf.<br>18. exp Reimbursement, Incentive/ OR (reimbursement incentive* OR financial incentive* OR economic incentive* OR monetary incentive* OR pay-for-performance OR P4P).ti,ab,kf.<br>19. "Cost Sharing"/ec OR Value-Based Health Insurance/ OR Value-Based Purchasing/ OR (((decreas* OR reduc* OR eliminat*) ADJ2 (copay* OR co-pay*)) OR value based insurance OR value based health insurance OR value based pricing OR value based benefit* OR VBBD OR VBID OR (full prescription ADJ2 coverage)).ti,ab,kf,hw.<br>20. OR/2-19<br>21. 1 AND 20<br>22. Limit 21 to (English language and yr="2000-current")<br>23. exp animals/ not exp humans/	

© 2024 Zhang et al. *JAMA Health Forum.*

	24. 22 NOT 23<br>25. Remove duplicates from 24	
**Embase (OVID) 1974-**	1. Exp Hypertension/ OR exp Antihypertensive Agent/ OR exp dipeptidyl carboxypeptidase inhibitor/ OR (hypertension OR hypertensive OR blood pressure OR antihypertensive* OR anti-hypertensive* OR beta blocker* OR diuretic* OR alpha blocker* OR vasodilator* OR ace inhibitor*).ti,ab,kf.<br>2. exp "Salary and Fringe Benefit"/ OR (minimum wage* OR hourly wage* OR hourly salary).ti,ab,kf.<br>3. (Insurance/ AND (Unemployment/ OR (unemploy* OR ((job OR employ*) ADJ2 loss) OR employment status).ti,ab,kf.)) OR (unemployment insurance OR unemployment benefit* OR unemployment coverage).ti,ab,kf.<br>4. (Earned Income Tax Credit OR EITC).ti,ab,kf.<br>5. ("Temporary Assistance for Needy Families" OR TANF OR "Aid to Families with Dependent Children" OR AFDC).ti,ab,kf.<br>6. Medical Leave/ OR (Paid leave OR Sick leave).ti,ab,kf.<br>7. Family Leave/ OR ("Family and Medical Leave Act" OR FMLA OR family leave OR medical leave).ti,ab,kf.<br>8. (ACA OR Affordable Care Act OR Obamacare).ti,ab,kf.<br>9. Exp Medicaid/ OR Medicaid Expansion.ti,ab,kf.<br>10. Exp Medicare/ OR ("Part D" OR (Medicare AND (drug coverage OR drug insurance OR drug benefit* OR (prescription* ADJ2 cost*))) OR (cap ADJ5 cost*) OR "Inflation Reduction Act" OR (medicare ADJ5 (savings OR beneficiary OR fee-for-service*)) OR "Qualifying Individual Program" OR "Qualified Disabled Working Individual Program").ti,ab,kf.<br>11. ((telehealth OR telemedicine OR tele-health OR tele-medicine OR ehealth OR mhealth OR mobile health OR audio only service*) ADJ5 (parity OR polic* OR law* OR regulation* OR payment* OR payer OR all-payer OR reimburse* OR waiver*)).ti,ab,kf.<br>12. (Housing/ AND (Public Policy/ OR health care policy/)) OR (Eviction Moratori* OR Eviction Protection* OR Housing Polic* OR Mortgage Relief OR Foreclosure Relief OR ((Home* OR housing) ADJ2 Afford*)).ti,ab,kf.<br>13. Food Assistance/ OR ("Supplemental Nutrition Assistance Program" OR SNAP OR "emergency supplement*" OR "Meals on Wheels" OR "Meal Replacement Benefits" OR "Nutrition Assistance" OR "Nutritional Assistance" OR "Women, Infants, and Children" OR WIC).ti,ab,kf.<br>14. "Economic Impact Payment*" OR "Stimulus Check*" OR "Inflation Relief Check*" OR "Child Tax Credit" OR "American Rescue Plan"	1201<br><br>- duplicates<br><br>=801 unique items

© 2024 Zhang et al. *JAMA Health Forum.*

	15. Social Security/ OR ("Supplemental Security Income" OR social security OR disability insurance).ti,ab,kf.<br>16. State Pharmaceutical Assistance Program*.ti,ab,kf.<br>17. (Economic* ADJ5 polic*).ti,ab,kf,hw.<br>18. exp economic incentive/ OR (reimbursement incentive* OR financial incentive* OR economic incentive* OR monetary incentive* OR pay-for-performance OR P4P).ti,ab,kf.<br>19. Value-Based Insurance design/ OR (((decreas* OR reduc* OR eliminat*) ADJ2 (copay* OR co-pay*)) OR value based insurance OR value based health insurance OR value based pricing OR value based benefit* OR VBBD OR VBID OR (full prescription ADJ2 coverage)).ti,ab,kf,hw.<br>20. OR/2-19<br>21. 1 AND 20<br>22. Limit 21 to (English language and yr="2000-current")<br>23. limit 22 to conference abstract status<br>24. 22 not 23<br>25. limit 24 to "remove medline records"<br>26. exp animal/ not exp human/<br>27. 25 NOT 26<br>28. Remove duplicates from 27	
**PsycInfo (OVID)**	1. (hypertension OR hypertensive OR blood pressure OR antihypertensive* OR anti-hypertensive* OR beta blocker* OR diuretic* OR alpha blocker* OR vasodilator* OR ace inhibitor*).ti,ab,sh,mh,hw,id.<br>2. (minimum wage* OR hourly wage* OR hourly salary).ti,ab,sh,mh,hw,id.<br>3. (unemployment insurance OR unemployment benefit* OR unemployment coverage).ti,ab,sh,mh,hw,id.<br>4. (Earned Income Tax Credit OR EITC).ti,ab,sh,mh,hw,id.<br>5. ("Temporary Assistance for Needy Families" OR TANF OR "Aid to Families with Dependent Children" OR AFDC).ti,ab,sh,mh,hw,id.<br>6. (Paid leave OR Sick leave).ti,ab,sh,mh,hw,id.<br>7. ("Family and Medical Leave Act" OR FMLA OR family leave OR medical leave).ti,ab,sh,mh,hw,id.<br>8. (ACA OR Affordable Care Act OR Obamacare).ti,ab,sh,mh,hw,id.<br>9. Medicaid Expansion.ti,ab,sh,mh,hw,id.<br>10. ("Part D" OR (Medicare AND (drug coverage OR drug insurance OR drug benefit* OR (prescription* ADJ2 cost*))) OR (cap ADJ5 cost*) OR "Inflation Reduction Act" OR (medicare ADJ5 (savings OR beneficiary OR fee-for-service*)) OR "Qualifying	223<br><br>- duplicates<br><br>=104 unique items

© 2024 Zhang et al. *JAMA Health Forum.*

	Individual Program" OR "Qualified Disabled Working Individual Program").ti,ab,sh,mh,hw,id.<br>11. ((telehealth OR telemedicine OR tele-health OR tele-medicine OR ehealth OR mhealth OR mobile health OR audio only service*) ADJ5 (parity OR polic* OR law* OR regulation* OR payment* OR payer OR all-payer OR reimburse* OR waiver*)).ti,ab,sh,mh,hw,id.<br>12. (Eviction Moratori* OR Eviction Protection* OR Housing Polic* OR Mortgage Relief OR Foreclosure Relief OR ((Home* OR housing) ADJ2 Afford*)).ti,ab,sh,mh,hw,id.<br>13. (food assistance OR "Supplemental Nutrition Assistance Program" OR SNAP OR "emergency supplement*" OR "Meals on Wheels" OR "Meal Replacement Benefits" OR "Nutrition Assistance" OR "Nutritional Assistance" OR "Women, Infants, and Children" OR WIC).ti,ab,sh,mh,hw,id.<br>14. "Economic Impact Payment*" OR "Stimulus Check*" OR "Inflation Relief Check*" OR "Child Tax Credit" OR "American Rescue Plan"<br>15. ("Supplemental Security Income" OR social security OR disability insurance).ti,ab,sh,mh,hw,id.<br>16. State Pharmaceutical Assistance Program*.ti,ab,sh,mh,hw,id.<br>17. (Economic* ADJ5 polic*).ti,ab,sh,mh,hw,id.<br>18. exp monetary Incentives/ OR (reimbursement incentive* OR financial incentive* OR economic incentive* OR monetary incentive* OR pay-for-performance OR P4P).ti,ab,sh,mh,hw.<br>19. (((decreas* OR reduc* OR eliminat*) ADJ2 (copay* OR co-pay*)) OR value based insurance OR value based health insurance OR value based pricing OR value based benefit* OR VBBD OR VBID OR (full prescription ADJ2 coverage)).ti,ab,sh,mh,hw.<br>20. OR/2-19<br>21. 1 AND 20<br>22. Limit 21 to (English language and yr="2000-current")	
**Cochrane Library**	#1 [mh "Hypertension"] OR [mh ^"Antihypertensive Agents"] OR [mh ^"Angiotensin-Converting Enzyme Inhibitors"] OR (hypertension OR hypertensive OR "blood pressure" OR antihypertensive* OR anti-hypertensive* OR (beta NEAR blocker*) OR diuretic* OR (alpha NEAR blocker*) OR vasodilator* OR (ace NEAR inhibitor*)):ti,ab,kw<br>#2 [mh "Salaries and Fringe Benefits"] OR ("minimum wage" OR "minimum wages" OR "hourly wage" OR "hourly wages" OR "hourly salary"):ti,ab<br>#3 ([mh "Insurance coverage"] AND ([mh "Unemployment"] OR (unemploy* OR ((job OR employ*) NEAR/2 loss) OR "employment status"):ti,ab)) OR ("unemployment insurance"	525<br><br>- duplicates<br><br>=265 unique items

© 2024 Zhang et al. *JAMA Health Forum.*

	OR "unemployment benefit" OR "unemployment benefits" OR "unemployment coverage"):ti,ab<br>#4 ("Earned Income Tax Credit" OR EITC):ti,ab<br>#5 [mh "Public Assistance"] OR [mh "Aid to Families with Dependent Children"] OR ("Temporary Assistance for Needy Families" OR TANF OR "Aid to Families with Dependent Children" OR AFDC):ti,ab<br>#6 [mh "Sick Leave"] OR ("Paid leave" OR "Sick leave"):ti,ab<br>#7 (FMLA OR "family leave" OR "medical leave"):ti,ab<br>#8 [mh "Patient Protection and Affordable Care Act"] OR (ACA OR "Affordable Care Act" OR Obamacare):ti,ab<br>#9 [mh "Medicaid"] OR "Medicaid Expansion":ti,ab<br>#10 [mh "Medicare"] OR [mh "Medicare Part D"] OR ("Part D" OR (Medicare AND ("drug coverage" OR "drug insurance" OR (drug NEAR benefit*) OR (prescription* NEAR/2 cost*))) OR (cap NEAR/5 cost*) OR "Inflation Reduction Act" OR (medicare NEAR/5 (savings OR beneficiary OR fee-for-service*)) OR "Qualifying Individual Program" OR "Qualified Disabled Working Individual Program"):ti,ab<br>#11 ((telehealth OR telemedicine OR tele-health OR tele-medicine OR ehealth OR mhealth OR "mobile health" OR "audio only service" OR "audio only services") NEAR/5 (parity OR polic* OR law* OR regulation* OR payment* OR payer OR all-payer OR reimburse* OR waiver*)):ti,ab<br>#12 ([mh "Housing"] AND [mh "Public Policy"]) OR (Eviction NEAR Moratori* OR Eviction NEAR Protection* OR Housing NEAR Polic* OR "Mortgage Relief" OR "Foreclosure Relief" OR ((Home* OR housing) NEAR/2 Afford*)):ti,ab<br>#13 [mh "Food Assistance"] OR ("food assistance" OR SNAP OR emergency NEAR supplement* OR "Meals on Wheels" OR "Meal Replacement Benefits" OR "Nutrition Assistance" OR "Nutritional Assistance" OR "Women, Infants, and Children" OR WIC):ti,ab<br>#14 ("Economic Impact Payment" OR "Economic Impact Payments" OR "Stimulus Check" OR "Stimulus Checks" OR "Inflation Relief Check" OR "Inflation Relief Checks" OR "Child Tax Credit" OR "American Rescue Plan"):ti,ab<br>#15 [mh "Social Security"] OR ("Supplemental Security Income" OR "social security" OR "disability insurance"):ti,ab<br>#16 [mh "Insurance, Pharmaceutical Services"] OR "State Pharmaceutical Assistance Programs":ti,ab<br>#17 (Economic* NEAR/5 polic*):ti,ab<br>#18 [mh ^"Reimbursement, Incentive"] OR (reimbursement NEAR incentive* OR financial NEAR incentive* OR economic NEAR incentive* OR monetary NEAR incentive* OR pay-for-performance OR P4P):ti,ab<br>#19 [mh "Cost Sharing"] OR [mh "Value-Based Health Insurance"] OR [mh "Value-Based Purchasing"] OR (((decreas* OR reduc*	

	OR eliminat*) NEAR/2 (copay* OR co-pay*)) OR "value based insurance" OR "value based health insurance" OR "value based pricing" OR ("value based" NEAR benefit*) OR VBBD OR VBID OR ("full prescription" NEAR/2 coverage)):ti,ab,kw<br>#20 OR/#2-#19<br>#21 #1 AND #20<br>Limit 2000 -	
**CINAHL (EBSCOHost)**	S1 (MH "Hypertension+") OR (MH "Antihypertensive Agents+") OR (MH "Angiotensin-Converting Enzyme Inhibitors+") OR (hypertension OR hypertensive OR "blood pressure" OR antihypertensive* OR anti-hypertensive* OR "beta blocker*" OR diuretic* OR "alpha blocker*" OR vasodilator* OR "ace inhibitor*")<br>S2 (MH "Salaries and Fringe Benefits") OR ("minimum wage*" OR "hourly wage*" OR "hourly salary") OR (MH "Insurance, Unemployment") OR ((MH "Insurance coverage") AND ((MH "Unemployment") OR (unemploy* OR ((job OR employ*) N2 loss) OR "employment status"))) OR ("unemployment insurance" OR "unemployment benefit*" OR "unemployment coverage") OR ("Earned Income Tax Credit" OR EITC) OR (MH "Public Assistance") OR (MH "Aid to Families with Dependent Children") OR ("Temporary Assistance for Needy Families" OR TANF OR "Aid to Families with Dependent Children" OR AFDC) OR (MH "Sick Leave") OR ("Paid leave" OR "Sick leave") OR (MH "Family and Medical Leave") OR (FMLA OR "family leave" OR "medical leave") OR (MH "Patient Protection and Affordable Care Act") OR (ACA OR "Affordable Care Act" OR Obamacare) OR (MH "United States Centers for Medicare and Medicaid Services") OR (MH "Medicaid") OR "Medicaid Expansion" OR (MH "Medicare") OR ("Part D" OR (Medicare AND ("drug coverage" OR "drug insurance" OR "drug benefit*" OR (prescription* N2 cost*))) OR (cap N5 cost*) OR "Inflation Reduction Act" OR (medicare N5 (savings OR beneficiary OR fee-for-service*)) OR "Qualifying Individual Program" OR "Qualified Disabled Working Individual Program") OR ((telehealth OR telemedicine OR tele-health OR tele-medicine OR ehealth OR mhealth OR "mobile health" OR "audio only service*") N5 (parity OR polic* OR law* OR regulation* OR payment* OR payer OR all-payer OR reimburse* OR waiver*)) OR ((MH "Housing") AND (MH "Public Policy")) OR ("Eviction Moratori*" OR "Eviction Protection*" OR "Housing Polic*" OR "Mortgage Relief" OR "Foreclosure Relief" OR ((Home* OR housing) N2 Afford*)) OR (MH "Food Assistance") OR ("food assistance" OR SNAP OR "emergency supplement*" OR "Meals on Wheels" OR "Meal Replacement Benefits" OR "Nutrition Assistance" OR "Nutritional Assistance" OR "Women, Infants, and Children" OR WIC) OR ("Economic Impact Payment*" OR	617<br><br>- duplicates<br><br>=291 unique items

© 2024 Zhang et al. *JAMA Health Forum.*

	"Stimulus Check*" OR "Inflation Relief Check*" OR "Child Tax Credit" OR "American Rescue Plan") OR ("Supplemental Security Income" OR "social security" OR "disability insurance") OR (MH "Insurance, Pharmaceutical Services") OR "State Pharmaceutical Assistance Programs" OR ((MH "Health policy") AND economic*) OR (Economic* N5 polic*) OR exp (MH "Reimbursement, Incentive") OR ("reimbursement incentive*" OR "financial incentive*" OR "economic incentive*" OR "monetary incentive*" OR pay-for-performance OR P4P) OR (MH "Cost Sharing") OR (MH "Value-Based Health Insurance") OR (MH "Value-Based Purchasing") OR (((decreas* OR reduc* OR eliminat*) N2 (copay* OR co-pay*)) OR "value based insurance" OR "value based health insurance" OR "value based pricing" OR "value based benefit*" OR VBBD OR VBID OR ("full prescription" N2 coverage))<br><br>S1 AND S2<br><br>**Limiters -** Published Date: 20000101-20231101; English Language; Exclude MEDLINE records	
**EconLit (EBSCOHost)**	S1 (hypertension OR hypertensive OR "blood pressure" OR antihypertensive* OR anti-hypertensive* OR "beta blocker*" OR diuretic* OR "alpha blocker*" OR vasodilator* OR "ace inhibitor*")<br><br>S2 ("minimum wage*" OR "hourly wage*" OR "hourly salary") OR (insurance AND (unemploy* OR ((job OR employ*) NEAR/2 loss) OR "employment status"))) OR ("unemployment insurance" OR "unemployment benefit*" OR "unemployment coverage") OR ("Earned Income Tax Credit" OR EITC) OR ("Temporary Assistance for Needy Families" OR TANF OR "Aid to Families with Dependent Children" OR AFDC) OR ("Paid leave" OR "Sick leave") OR (FMLA OR "family leave" OR "medical leave") OR (ACA OR "Affordable Care Act" OR Obamacare) OR "Medicaid Expansion" OR ("Part D" OR (Medicare AND ("drug coverage" OR "drug insurance" OR "drug benefit*" OR (prescription* N2 cost*))) OR (cap N5 cost*) OR "Inflation Reduction Act" OR (medicare N5 (savings OR beneficiary OR fee-for-service*)) OR "Qualifying Individual Program" OR "Qualified Disabled Working Individual Program") OR ((telehealth OR telemedicine OR tele-health OR tele-medicine OR ehealth OR mhealth OR "mobile health" OR "audio only service*") N5 (parity OR polic* OR law* OR regulation* OR payment* OR payer OR all-payer OR reimburse* OR waiver*)) OR (Housing AND "Public Policy") OR ("Eviction Moratori*" OR "Eviction Protection*" OR "Housing	136<br><br>- duplicates<br><br>=104 unique items

© 2024 Zhang et al. *JAMA Health Forum.*

	Polic*" OR "Mortgage Relief" OR "Foreclosure Relief" OR ((Home* OR housing) N2 Afford*)) OR ("food assistance" OR SNAP OR "emergency supplement*" OR "Meals on Wheels" OR "Meal Replacement Benefits" OR "Nutrition Assistance" OR "Nutritional Assistance" OR "Women, Infants, and Children" OR WIC) OR ("Economic Impact Payment*" OR "Stimulus Check*" OR "Inflation Relief Check*" OR "Child Tax Credit" OR "American Rescue Plan") OR ("Supplemental Security Income" OR "social security" OR "disability insurance") OR "State Pharmaceutical Assistance Programs" OR (Economic* N5 polic*) OR ("reimbursement incentive*" OR "financial incentive*" OR "economic incentive*" OR "monetary incentive*" OR pay-for-performance OR P4P) OR (((decreas* OR reduc* OR eliminat*) N2 (copay* OR co-pay*)) OR "value based insurance" OR "value based health insurance" OR "value based pricing" OR "value based benefit*" OR VBBD OR VBID OR ("full prescription" N2 coverage))<br><br>**Limiters** - Published Date: 20000101-20231101; English Language;	
**Sociological Abstracts (ProQuest)**	TI,AB,SU(hypertension OR hypertensive OR "blood pressure" OR antihypertensive* OR anti-hypertensive* OR "beta blocker*" OR diuretic* OR "alpha blocker*" OR vasodilator* OR "ace inhibitor*")<br>AND<br>("minimum wage*" OR "hourly wage*" OR "hourly salary" OR (insurance AND (unemploy* OR (job loss OR (employ* NEAR/2 loss) OR "employment status"))) OR "unemployment insurance" OR "unemployment benefit*" OR "unemployment coverage" OR "Earned Income Tax Credit" OR EITC OR "Temporary Assistance for Needy Families" OR TANF OR "Aid to Families with Dependent Children" OR AFDC OR "Paid leave" OR "Sick leave" OR FMLA OR "family leave" OR "medical leave" OR ACA OR "Affordable Care Act" OR Obamacare OR "Medicaid Expansion" OR "Part D" OR (Medicare AND ("drug coverage" OR "drug insurance" OR "drug benefit*" OR (prescription* NEAR/2 cost*))) OR (cap NEAR/5 cost*) OR "Inflation Reduction Act" OR (medicare NEAR/5 (savings OR beneficiary OR fee-for-service*)) OR "Qualifying Individual Program" OR "Qualified Disabled Working Individual Program" OR ((telehealth OR telemedicine OR tele-health OR tele-medicine OR ehealth OR mhealth OR "mobile health" OR "audio only service*") NEAR/5 (parity OR polic* OR law* OR regulation* OR payment* OR payer OR all-payer OR reimburse* OR waiver*)) OR (Housing AND "Public Policy") OR "Eviction Moratori*" OR "Eviction Protection*" OR "Housing Polic*" OR "Mortgage Relief" OR "Foreclosure Relief" OR ((Home* OR housing) NEAR/2 Afford*) OR "food assistance" OR SNAP OR "emergency supplement*" OR "Meals on Wheels" OR	311<br><br>- duplicates<br><br>=259 unique items

© 2024 Zhang et al. *JAMA Health Forum.*

	"Meal Replacement Benefits" OR "Nutrition Assistance" OR "Nutritional Assistance" OR "Women, Infants, and Children" OR WIC OR "Economic Impact Payment*" OR "Stimulus Check*" OR "Inflation Relief Check*" OR "Child Tax Credit" OR "American Rescue Plan" OR "Supplemental Security Income" OR "social security" OR "disability insurance" OR "State Pharmaceutical Assistance Programs" OR (Economic* NEAR/5 polic*) OR "reimbursement incentive*" OR "financial incentive*" OR "economic incentive*" OR "monetary incentive*" OR pay-for-performance OR P4P) OR (((decreas* OR reduc* OR eliminat*) NEAR/2 (copay* OR co-pay*)) OR "value based insurance" OR "value based health insurance" OR "value based pricing" OR "value based benefit*" OR VBBD OR VBID OR ("full prescription" NEAR/2 coverage))<br>Limit English; 2000 - current	
**Scopus**	TITLE-ABS-KEY(hypertension OR hypertensive OR "blood pressure" OR antihypertensive* OR anti-hypertensive* OR "beta blocker*" OR diuretic* OR "alpha blocker*" OR vasodilator* OR "ace inhibitor*") AND TITLE-ABS-KEY("minimum wage*" OR "hourly wage*" OR "hourly salary" OR (insurance AND (unemploy* OR (job loss OR (employ* W/2 loss) OR "employment status"))) OR "unemployment insurance" OR "unemployment benefit*" OR "unemployment coverage" OR "Earned Income Tax Credit" OR EITC OR "Temporary Assistance for Needy Families" OR TANF OR "Aid to Families with Dependent Children" OR AFDC OR "Paid leave" OR "Sick leave" OR FMLA OR "family leave" OR "medical leave" OR ACA OR "Affordable Care Act" OR Obamacare OR "Medicaid Expansion" OR "Part D" OR (Medicare AND ("drug coverage" OR "drug insurance" OR "drug benefit*" OR (prescription* W/2 cost*))) OR (cap W/5 cost*) OR "Inflation Reduction Act" OR (medicare W/5 (savings OR beneficiary OR fee-for-service*)) OR "Qualifying Individual Program" OR "Qualified Disabled Working Individual Program" OR ((telehealth OR telemedicine OR tele-health OR tele-medicine OR ehealth OR mhealth OR "mobile health" OR "audio only service*") W/5 (parity OR polic* OR law* OR regulation* OR payment* OR payer OR all-payer OR reimburse* OR waiver*)) OR (Housing AND "Public Policy") OR "Eviction Moratori*" OR "Eviction Protection*" OR "Housing Polic*" OR "Mortgage Relief" OR "Foreclosure Relief" OR ((Home* OR housing) W/2 Afford*) OR "food assistance" OR SNAP OR "emergency supplement*" OR "Meals on Wheels" OR "Meal Replacement Benefits" OR "Nutrition Assistance" OR "Nutritional Assistance" OR "Women, Infants, and Children" OR WIC OR "Economic Impact Payment*" OR "Stimulus Check*" OR "Inflation Relief Check*" OR "Child Tax Credit" OR "American Rescue Plan" OR "Supplemental Security Income" OR "social security" OR "disability insurance" OR "State Pharmaceutical Assistance	794<br><br>- duplicates<br><br>=248 unique items

	Programs" OR (Economic* W/5 polic*) OR "reimbursement incentive*" OR "financial incentive*" OR "economic incentive*" OR "monetary incentive*" OR pay-for-performance OR P4P OR (((decreas* OR reduc* OR eliminat*) W/2 (copay* OR co-pay*)) OR "value based insurance" OR "value based health insurance" OR "value based pricing" OR "value based benefit*" OR VBBD OR VBID OR ("full prescription" W/2 coverage))) AND NOT INDEX(medline)<br>Limit English; 2000 - current	

Notes: Duplicates were identified using the Endnote automated "find duplicates" function with preference set to match on title, author and year, and removed from your Endnote library. There will likely be additional duplicates found that Endnote was unable to detect.

eTable 2. Inclusion and Exclusion Criteria

**Inclusion criteria**
• Economic policies in the United States at different levels (federal, state, local policies)<br>• Published between 2000 and 2023<br>• Published in English language only<br>• Peer-reviewed empirical studies only<br>• Study population can include pregnant women
**Exclusion criteria**
• Study population with children<br>• Descriptive studies, simulation studies (based on secondary data analysis and a lot of assumptions), grey literature (reports, working papers, white papers), dissertations

eTable 3. Bias Assessment Checklist and Rubric

Bias category	Score range	Scoring rubric
**Bias due to confounding**	**(0-3)**	
Having controls at the level of unit of analysis	(0-1)	1: If the study controlled for all measured individual-level confounders<br>0: If the study did not control for individual-level confounders or failed to control for a known confounder
Having contextual-level controls	(0-1)	1: Controlled for fixed effects or confounders at levels above or beyond the individual level (eg, state, county, or community levels)<br>0: Otherwise
Having time controls	(0-1)	1: Controlled for the time variable (eg, year, quarter, month) or secular trends<br>0: Otherwise
**Bias due to selection**	**(0-2)**	
Selection of participants into the study reflect the target population	(0,1)	1: No selection bias present<br>0: Selection bias present: (1) a nationwide policy analysis fails to account for sampling weights provided

© 2024 Zhang et al. *JAMA Health Forum.*

		in a nationally representative survey; (2) the study used a convenient sample to study a policy's impact but claimed an effect for the target population of interest; (3) participants' self-selection into the treatment group led to systematic differences between the treatment and comparison groups; or (4) other sample selection issues (for other sample selection issues, recommend documenting what the issue is as data being extracted).
Selection of participants into the study reflect the target population	(0,1)	1: (1) Missing data < 5%; (2) missing data ≥ 5% and was handled appropriately (ie, reported missing data or attrition/loss-to-follow-up rate, provided rationale for not addressing missing data; mentioned and used statistical approaches to address missing data issues, and used an appropriate method to address the missing value problems)<br>0: (1) The study did not report missing data or attrition/loss-to-follow-up rate; (2) missing data ≥ 5% but did not use statistical approaches to address missing data issues or did not provide rationale for not addressing them; (3) missing data ≥ 5% but used a method that was inappropriate to address the missing value problems (eg, missing not at random but used multiple imputations) in the data
**Bias in measurement of outcomes**	**(1-3)**	
Medication adherence measured by the electronic drug monitoring approach; or BP measured by a certified health care professional; or self-measured BP using clinically validated devices	(0,3)	3: Electronic drug monitors, including MEMS, consist of specialized microchips incorporated into medication bottles that catalogue every opening of the bottle. It is sometimes used as a gold standard to measure medication adherence. BP measures are considered reliable and valid if they are measured using clinically validated devices by clinicians or consumers. A full list of clinically validate devices is provided here: https://www.nature.com/articles/s41371-022-00718-5/tables/1
Measured BP in claims or EHRs; or prescription of antihypertensive medications measured by EHRs or pharmacy fill/refill data	(0,2)	2: The utilization of pharmacy fill/refill data does not require patient participation or provide objective measurement of medication adherence. Therefore, the medication adherence value obtained from fill/refill data does not produce any information on medication consumption; rather, it solely provides assessment of acquisition and possession of medication.
Self-reported measures on currently taking antihypertensive medications; or self-reported BP measures with no mention of devices or settings	(0,1)	1: Self-reported BP or self-reported medication use is less reliable because it is subject to both measurement error and recall bias
**Bias in reported results**	**(0-2)**	

© 2024 Zhang et al. *JAMA Health Forum.*

All pre-specified outcomes were reported	(0,1)	This criterion refers to the study's potential publication bias:<br>1: Reported findings on all outcomes of interest (eg, primary and secondary outcomes in the Methods), including outcomes in sensitivity analyses and supplementary materials<br>0: Otherwise
Funding sources and potential conflicts of interest were reported	(0,1)	This criterion assesses the possibility that the study's findings were biased by sources of funding or conflicts of interest:<br>1: Reported no conflict of interest<br>0: (1) Potential conflicts of interest reported; (2) reported funding sources that may compromise an investigator's professional judgment in reporting the research
**Overall quality score**	**(1-10)**	This criterion refers to the study's overall quality score:<br>8-10: Good (contains low risk of bias)<br>5-7: Moderate (contains moderate risk of bias)<br>1-4: Poor (contains high risk of bias)

Abbreviations: BP, blood pressure; MEMS, medication event monitoring system; EHR, electronic health records.

© 2024 Zhang et al. *JAMA Health Forum.*

eTable 4. Evidence Extraction Main Table

Study	Population	Study design	Analytical models	Measures of hypertension-related outcome
Fischer, 2007	Medicaid recipients with the traditional fee-for-service plans who were prescribed antihypertensive medications	Quasi-experimental	Interrupted time series analysis with general linear models, using generalized estimating equations to adjust for repeated observations	The proportion of ARB units dispensed among the total RAAS-blocking medications (ACE inhibitor and ARB) used quarterly by each state Medicaid program
Maciejewski, 2010	A convenient sample of veterans with hypertension who were diagnosed and prescribed a medication at 4 large tertiary VA medical centers	Quasi-experimental	Logistic regressions to conduct the 1-to-1 nearest-neighbor propensity score matching with replacement; generalized estimating equations with person-month as the unit of analysis	Adherence to antihypertensive medications using the validated ReComp algorithm based on the proportion of days covered. Subjects were considered adherent if they had medications available for at least 80% of each month.
Zhang, 2010	Medicare beneficiaries with hypertension enrolled in Medicare Advantage plans	Quasi-experimental	Logistic regression to conduct propensity score weighting; generalized linear model for the regression on Medication Possession Ratio (MPR) measure and treatment intensity; logistic regression for the good medication adherence measure (MPR $\geq$ 0.80)	Continuous MPR; a categorical measure of good adherence measured by counts of average daily medication use; a measure of treatment intensity
Zhang, 2011	Medicare beneficiaries—enrolled in Medicare Advantage plans sold by a large insurance company in Pennsylvania—who had at least 2 claims in 2003 with a diagnosis coded for hypertension	Quasi-experimental	Logistic regressions were used to conduct propensity score weighting; general estimating equations adjusted for correlations in repeated measures within individuals	Average daily counts of any antihypertensive filled each year. Proportion using ARBs over less expensive ACE inhibitors.
Li, 2012	Medicare beneficiaries aged 65 years or older covered under fee-for-service Medicare with diagnoses for hypertension	Quasi-experimental	Segmented regression models that used generalized estimating equations with first-order, autoregressive correlation structure; 2-period, difference-in-differences (DiD) logistic regression models with the generalized estimating equation method	Use of antihypertensive medications were measured by number of 30-day supply-equivalent prescriptions available per month, medication adherence, and continuous medication gaps

© 2024 Zhang et al. *JAMA Health Forum.*

Wang, 2013	Medicaid patients with diagnosis of hypertension—in Louisiana	Quasi-experimental	Cox proportional hazard models analyzed the discontinuation rate of antihypertensive prescriptions	Discontinuation of therapy was measured as patients without available medication for more than 30 consecutive days
Baicker, 2013	Low-income, uninsured, able-bodied adults aged 19 to 64 years and Oregon residents who were U.S. citizens or legal immigrants and are not eligible for other public insurance in Oregon	Randomized controlled trial	Linear probability models for binary outcomes; logistic regressions as sensitivity checks	Systolic and diastolic blood pressure; Current use of medication for hypertension
Bardach, 2013	Clinic eligibility criteria were having at least 200 patients eligible for measurement, having at least 10% Medicaid or uninsured patients, and use of the EHR software for at least 3 months.	Cluster randomized controlled trial	Multilevel mixed-effects logistic regression to model patient-level measure performance. Sensitivity analyses to address potential bias due to postrandomization drop out.	Blood pressure measure
Petersen, 2013	VA patients with hypertension	Cluster randomized controlled trial	Chi-square tests for binomial variables (or Fisher exact test when cell sizes were <5) and the Kruskal-Wallis rank test for continuous variables. A repeated-measures longitudinal analysis using the hospital as a random effect.	Blood pressure control or appropriate response to uncontrolled blood pressure; Use of guideline-recommended antihypertensive medications
Zimmer, 2014	Medicare beneficiaries aged 65-74 years	Quasi-experimental	Zero-inflated negative binomial model estimated counts of prescriptions (including refills)	Number of prescriptions for hypertension
Amin, 2017	Medicaid enrollees taking antihypertensive medications—in North Carolina	Quasi-experimental	A partial difference-in-difference-in-differences (DDD) method with individual-level fixed effects; sensitivity analyses	Medication adherence (proportion of days covered [PDC]) was stratified into 3 groups: fully adherent (PDC ≥ 80%), partially adherent (50%-79%), and nonadherent (PDC ≤ 50%)
Cole, 2017	Patients who received health care from community health centers (CHCs) that had received grants under section 330	Quasi-experimental	A DiD analysis with inverse probability of treatment weights; all observations were clustered at the health center level to account for repeated measures; sensitivity analyses	Hypertension control rate (BP < 140/90 mm Hg) for each CHC

© 2024 Zhang et al. *JAMA Health Forum.*

Hatch, 2017	OCHIN (a non-profit network of CHCs) patients with uncontrolled hypertension	Quasi-experimental	Propensity score-matching; Cox proportional hazards model; linear mixed effect models; generalized estimating equation logistic regression model	(1) Time from baseline uncontrolled hypertension to a follow-up controlled hypertension; (2) systolic and diastolic BP; (3) having a disease-specific medication ordered
Kostova, 2017	Low-income persons aged 20–64 who were identified to have hypertension who were enrolled in Medicaid, and persons not enrolled in Medicaid who had family incomes at or below 250% of the federal poverty level	Quasi-experimental	A differencing regression model was used to evaluate health outcomes among Medicaid beneficiaries	Uncontrolled hypertension; self-reported currently taking antihypertensive drugs
Adams, 2017	Medicare and Medicaid dually eligible patients with at least one inpatient or two outpatient diagnoses of common cancers (breast, colorectal, prostate, lung, or cervical cancer)	Quasi-experimental	An Interrupted Time Series analysis to control for auto-correlation and pre-existing trends in medication use	1) Prevalence of antihypertensive medication use (proportion of patients with medication available during the month on the basis of current and previous dispensing per therapeutic drug class per month and 2) intensity of medication use, calculated as the number of days' supply dispensed per therapeutic drug class per month across the entire cohort
Hirth, 2017	Connecticut state employees and their dependents ages 18–64 who were continuously enrolled in the state's employer-sponsored insurance plan	Quasi-experimental	A difference-in-differences framework with linear probability models to estimate binary outcomes, and ordinary least squares models for the continuous spending outcomes	Adherence to antihypertensive medications was measured by medication possession ratios (MPR), calculated as the percentage of days in each year for which a filled prescription was available
McWilliams, 2017	Medicare fee-for-service beneficiaries who were using antihypertensives	Quasi-experimental	A DiD approach and linear regression; sensitivity analyses	(1) Any use (at least 1 prescription fill) and PDC for ACE inhibitors and ARBs, β-blockers, thiazide diuretics, calcium channel blockers separately; (2) a dichotomous indicator of adherence (PDC $\geq$ 80%)

© 2024 Zhang et al. *JAMA Health Forum.*

Cole, 2018	Patients who received health care from CHCs that had received grants under section 330	Quasi-experimental	A DiD analysis with inverse probability of treatment weights; generalized linear models that assumed a negative binomial distribution with log link; sensitivity analyses	Hypertension control rate (BP < 140/90 mm Hg) for each CHC
Diebold, 2018	Noninstitutionalized Medicare beneficiaries, excluding beneficiaries with prescription coverage through Medicaid, VA, or TRICARE	Quasi-experimental	A DiD analysis using logistic regression for binary outcome and ordinal logistic regression for ordinal outcome	A 3-category dependent variable that indicates whether the high BP of hypertensive respondents is better (HBPUC = 1), about the same (HBPUC = 2), or worse (HBPUC = 3) than it was in the previous wave
Melissa, 2018	Medicaid enrollees with chronic conditions in 6 states	Quasi-experimental	A DiD approach with logistic regression models for the binary outcome	Participants lowered their BP to < 140 mm Hg from baseline BP of ≥ 140 mm Hg
Kaboli, 2018	All patients who received primary care at 13 Veterans Affairs outpatient clinics affiliated with the Iowa City and Minneapolis Veterans Affairs Medical Centers, with hypertension who were not taking a thiazide and were not at an appropriate BP goal at the 2 most recent clinic visits	Randomized controlled trial	Hierarchical logistic regression using the generalized linear mixed model procedure.	Thiazide prescribing and BP control
Angier, 2020	Medicaid expansion eligible patients aged 19-64 years diagnosed with hypertension prior to ACA Medicaid expansion implementation (pregnant women were excluded)	Quasi-experimental	Logistic mixed effects models using random intercepts for patients and clinics and a random slope for year accounted for temporal correlation of observations within patients and clustering of patients within clinics	Controlled hypertension defined as a binary indicator of whether a patient's BP was within a normal range: < 140 mm Hg SBP and < 90 mm Hg DBP
Margerison, 2020	Women of reproductive age (18-44 years) with low household income (< 138% federal poverty level) and were not pregnant	Quasi-experimental	Multivariable, linear probability DiD models with robust SEs clustered by state	Currently taking BP medication

© 2024 Zhang et al. *JAMA Health Forum.*

Marino, 2020	Patients aged 19-64 years who were diagnosed with diabetes prior to the Medicaid expansion	Quasi-experimental	A DiD analysis using a linear mixed-effects model	SBP and DBP
Cole, 2021	Patients with hypertension aged 18-85 years who received services from the Health Resources and Services Administration (HRSA)-funded Federally Qualified Health Centers (FQHCs)	Quasi-experimental	A DiD approach using linear probability models	Proportion of patients with hypertension with BP < 140/90 mm Hg
Gotanda, 2021	Individuals aged 19-64 years with family incomes < 138% of the federal poverty level	Quasi-experimental	A DiD method using multivariable linear regression models	Mean SBP and DBP were calculated after excluding the first reading of individuals with more than 1 value
Kim, 2021	Nonelderly individuals	Quasi-experimental	DiD linear regression models	The length of time since the patients last took their BP medicine
Lanese, 2021	Individuals and families facing homelessness and receiving primary care and related services from the Health Care for the Homeless (HCH) projects	Quasi-experimental	Linear mixed models were utilized with DiD estimation	Individuals ages 18-85 years diagnosed with hypertension and BP sufficiently controlled (< 140/90 mm Hg) during the measurement year
Peterson, 2021	Medicare patients enrolled in the Million Hearts Model in 2017 who had medium or high CVD risk at enrollment and who had Part D coverage	Cluster randomized controlled trial	Logistic regressions to estimate model effects on initiation or intensification of antihypertensive medications and linear regressions to measure associations with BP	Whether patients with clinical risk factors initiated or intensified antihypertensive therapy within a year of enrolling in the model; and mean SBP
Sumarsono, 2021	Medicaid beneficiaries among all 50 states and Washington DC with cardiovascular diseases	Quasi-experimental	A difference-in-differences (DiD) analysis in the average quarterly prescription rates (per 1000 Medicaid beneficiaries) for these cardiovascular therapies	Number of quarterly filled prescriptions for antihypertensive medications per 1000 Medicaid beneficiaries

© 2024 Zhang et al. *JAMA Health Forum.*

Fakeye, 2022	Medicaid insured and privately insured beneficiaries in Maryland	Quasi-experimental	A DiD analytic approach using the hierarchical linear regression model	Semiannual medication possession ratio, a measure that tracks gaps in medication adherence by aggregating days' supply of antihypertensive prescriptions over a defined period of a calendar half year

eTable 4 (Cont.) Evidence Extraction Main Table

		Time Horizon		Experimental groups	
**Study**	**Data source**	**Pre-policy**	**Post-policy**	**Economic policy**	**Comparison**
Fischer, 2007	Centers for Medicare and Medicaid Services (CMS), State Drug Utilization Data, 2005	1st quarter of 1996 to/by the 3rd quarter of 2004	4th quarter of 2004 to the 2nd quarter of 2005	Medicaid prior authorization (PA) on costly ARBs; type of PA program (a preferred drug list [PDL] only vs ACE inhibitor trial required) by the 3rd quarter of 2004	Medicaid without prior authorization by the 3rd quarter of 2004
Maciejewski, 2010	4 VA medical centers datasets for 2001-2003	February 2001 to January 2002	February 2002 to January 2003 (short-term post period) and February 2003 to December 2003 (longer-term post period)	Systemwide increase in the VA medication copayment from $2 to $7 in February 2002 for non-exempt veterans	Propensity-matched veterans who were exempt from medication copayments due to low income or military service-related disability
Zhang, 2010	A 40% random sample of Medicare beneficiaries who were enrolled with Medicare Advantage insurers between January 2003 and December 2007	January 2004 to December 2005	January 2006 to December 2007	3 Medicare Part D interventions: no drug coverage ("no-coverage group"); relatively poor drug coverage with a $150 quarterly cap in plan payment ("$150-cap group"); relatively good coverage with a $350 quarterly cap ("$350-cap group")	Medicare Advantage plans with no drug cap
Zhang, 2011	A 40% random sample of Medicare beneficiaries who were enrolled with Medicare Advantage insurers between January 2003 and December 2007	January 2004 to December 2006	January 2006 to December 2008	(1) No previous drug coverage (no coverage); (2) poor previous drug coverage (US $150 quarterly cap of plan payment [US $150 cap]); (3) drug coverage that is comparable to that offered under Part D (US $350 cap)	Generous employer-sponsored drug coverage

© 2024 Zhang et al. *JAMA Health Forum.*

Li, 2012	Medicare inpatient, outpatient, and carrier claims files from 2005 and 2006 for a 5% random sample of Medicare beneficiaries, linked to the 2006 Medicare Part D prescription drug event file and a Part D plan characteristics file	February 2006 to 1 month before coverage gap entry	The month of coverage gap entry to 1 month before catastrophic coverage entry, or December 2006, whichever came earlier	3 coverage gaps: (1) 100% cost sharing for any drug; (2) generic-only coverage (100% cost sharing for only brand-name drugs); (3) brand-name and generic coverage (no change in cost sharing for any drug)	Full low-income subsidies during the coverage gap
Wang, 2013	2001-2003 Medicaid Analytic Extract files for Louisiana and Indiana, maintained by CMS	6 months prior to both the pre-policy (March 2002) and policy period (March 2003)	March 3, 2003, or March 3, 2002, until medication discontinuation or the end of the pre-policy or policy period year	Medicaid program in Louisiana that adopted a monthly prescription limit	Medicaid program in Indiana that had the same level of copayments but did not enforce a monthly prescription limit policy
Baicker, 2013	Data-collection protocol included detailed questionnaires and performance of anthropometric and blood pressure measurements. Dried blood spots were also obtained.	2008	September 2009 and December 2010	Medicaid coverage through a lottery drawing	Selected adults who did not win the lottery for Medicaid coverage
Bardach, 2013	Final documentation in the EHR from the study period	March, 2009	April 2009 to March 2010	Pay-for-performance: receiving financial incentives and benchmarked quarterly reports of their performance	Receiving only quarterly reports
Petersen, 2013	Electronic health records for 40 patients with hypertension randomly selected from each physician's panel	August-November 2007	April, 2010, and 12-month follow-up	(1) physician-level (individual) incentives; (2) practice-level incentives; (3) physician-level plus practice-level (combined) incentives	No incentives
Zimmer, 2014	Medical expenditure panel survey 2000-2004 and 2006-2008	2000-2004 (the years prior to Part D)	2006-2008 (3 years after Part D)	Medicare Part D for the elderly population	Near-elderly population aged 56-64 who did not qualify for Medicare Part D
Amin, 2017	Medicaid claims and enrollment data from CMS for North Carolina and Georgia	Prechange Period January 1, 2000-June 3, 2001	Transition Period July 1, 2001-September 31, 2001, and Postperiod October 1, 2001-December 31, 2002	North Carolina Medicaid program policies of restrictions on days' supply and increased copayment for brand-name medications	(1) North Carolina Medicaid beneficiaries who were not likely to be affected by the policy changes; (2) Georgia Medicaid enrollees who

© 2024 Zhang et al. *JAMA Health Forum.*

					were not prescribed long-term prescriptions
Cole, 2017	Uniform Data System 2011-2014 collected annually by HRSA's Bureau of Primary Health Care and are reported at the health center level	2011-2013	2014	States that expanded eligibility for Medicaid to all nonelderly adult citizens and qualifying permanent residents with incomes up to 138% of the federal poverty level	States that had not expanded Medicaid eligibility as of January 2014
Hatch, 2017	OCHIN EHR, linked with Medicaid enrollment data from the state of Oregon (2008-2011)	0 to 6 months before Medicaid expansion in Oregon	6-24 months after Medicaid expansion	Oregon's Medicaid expansion	A propensity score matched sample in Oregon that were continuously uninsured
McWilliams, 2017	Medicare claims and enrollment files for a random 20% sample of fee-for-service beneficiaries continuously enrolled in Parts A, B, and D in that year (while alive for decedents) and in the previous year from 2009 to 2014, and the CMS Accountable Care Organizations (ACOs) Provider-Level Research Identifiable File	(1) 2009-2011 for ACOs entering the Medicare Shared Savings Program (MSSP) in 2012; (2) 2009-2012 for ACOs entering in 2013; (3) 2009-2013 for ACOs entering in 2014	Non-ACO providers	Participation of accountable care organizations in MSSP	Local non-ACO providers
Kostova, 2017	National Health and Nutrition Examination Survey 1999–2012	1999–2002	2009–2012	Medicaid beneficiaries in 4 states that introduced copayments during the study period	Medicaid beneficiaries in 14 states unaffected by shifts in copayment policy, and a within-state counterfactual group of low-income adults not on Medicaid

© 2024 Zhang et al. *JAMA Health Forum.*

Adams, 2017	Data from a 5 nationally representative sample of linked Medicaid, Medicare, and PartD drug claims for dual enrollees for the years 2004 through 2007 provided by the Centers for Medicare & Medicaid Services	2004-2005	2006-2007	Transition from Medicaid drug caps on the number of medications reimbursed per month to Medicare Part D that prohibits the use of caps	Transition from Medicaid in non-capped states to Medicare Part D
Hirth, 2017	Data for the intervention group were from the claims data for Connecticut state employees and their dependents. Data for the comparison group were obtained from a sample of state government employees and dependents from the Truven MarketScan Commercial Claims and Encounters Database for the same period. That sample included employees of six state employers and their dependents.	July 1, 2010-October 1, 2011	October 1, 2011-June 30, 2013	Connecticut implemented a value-based insurance design (VBID, the Health Enhancement Program) for state employees in 2011 to encourage consumers to use high-value medical services	State employees of other states that did not have a VBID program
Cole, 2018	Uniform Data System for 2011-2015 or 2012-2015 collected annually by the Bureau of Primary Health Care of the HRSA on CHCs	2011/2012-2013	2014-2015	Rural or urban CHCs in states that expanded eligibility for Medicaid by 2014	Rural or urban CHCs in states that had not expanded Medicaid by 2014
Diebold, 2018	Data used in this study come from the 2000-2010 waves of the Health and Retirement Study (HRS), a biennial longitudinal survey that follows	2000-2004	2006-2010	Medicare Part D	Beneficiaries who previously had and maintained prescription coverage from a private provider outside of the Medicare program

© 2024 Zhang et al. *JAMA Health Forum.*

	nationally representative cohorts of older Americans				
Melissa, 2018	Medicaid claims data from 6 states covering 2 years before the start of the state's Medicaid Incentives for Prevention of Chronic Diseases (MIPCD) program through December 2015	2011-2013	2013-2015	The 2011 ACA authorized the MIPCD program ($135($90) for process only incentives)	A control group that did not receive financial incentives
Angier, 2020	EHR data from the Accelerating Data Value Across a National Community Health Center Network (ADVANCE) clinical data research network from 5 states that expanded Medicaid eligibility on 1/1/2014 (California, Ohio, Oregon, Washington, Wisconsin)	January 1, 2012-December 31, 2013	January 1, 2014-December 31, 2017	Newly insured or continuously insured under the Medicaid expansion policy	Continuously uninsured or discontinuously insured groups during the study period
Margerison, 2020	Behavioral Risk Factor Surveillance System 2011-2016	2011-2013	2015-2016	States that expanded Medicaid by January 1, 2014, excluded 5 states that expanded Medicaid between January 2014 and the end of 2015, and 7 states that had partial Medicaid expansions prior to 2014	States that did not expand Medicaid
Marino, 2020	Patient-level EHR data were derived from the ADVANCE clinical data research network (CDRN) of PCORnet, which contains data from CHCs in 10 states that expanded Medicaid	January 1, 2012-December 31, 2013	January 1, 2014-December 31, 2015	Newly insured or continuously insured in the 10 states that expanded Medicaid as of January 1, 2014 (California, Hawaii, Maryland, Minnesota, New Mexico, Ohio, Oregon, Rhode Island, Washington, Wisconsin)	Continuously uninsured or discontinuously insured in the same 10 states

© 2024 Zhang et al. *JAMA Health Forum.*

Cole, 2021	HRSA 2012-2018 Uniform Data System, a sample comprising all HRSA-funded FQHCs that includes annual data on patient characteristics, organizational and structural characteristics, quality of care measures, and service volume	2012-2013	2014-2018	Medicaid expansion in 2014	States that had not expanded Medicaid by 2018
Gotanda, 2021	2005-2016 National Health and Nutrition Examination Survey	2005-2012	2015-2016	Medicaid expansion on January 1, 2014	States that did not expand Medicaid
Kim, 2021	Behavioral Risk Factor Surveillance System 2011-2016	2011-2013	2015-2016	Medicaid expansion in 2014	States that did not expand Medicaid
Lanese, 2021	Uniform Data System for health centers through a Freedom of Information Act request fulfilment and direct contact with The National Health Care for the Homeless (HCH) Council; only HCHs were included as defined by HRSA and Department of Health and Human Services	2012-2014	2015-2019	State Medicaid expansion status as of 2015	States that did not expand Medicaid
Peterson, 2021	Data from the organizations that participated in the Million Hearts CVD Risk Reduction Model launched by CMS in 2017	Baseline: 2017	Follow-up: 2018	In 2017, CMS paid intervention organizations $10 per high-risk patient per month for care management services. Starting in 2018, CMS paid $0-$10 per high-risk patient per month depending on the organization's success in reducing the mean risk score among high-risk patients ($0 for a mean decline of < 2 percentage points, $5 for a 2- to 10-point decline, and $10 for > 10-point decline). CMS sent intervention organizations semiannual reports describing performance enrolling	CMS paid control organizations that submitted clinical data ($20 per patient per submission) that CMS used to calculate Medicare patients' CVD risk—at enrollment and annually through 2019. CMS did not report CVD risk scores to control organizations or ask organizations to calculate risk scores themselves.

© 2024 Zhang et al. *JAMA Health Forum.*

				patients and reducing CVD risk. CMS also offered quarterly peer-to-peer learning sessions.	
Sumarsono, 2021	Medicaid State Drug Utilization Dataset and Current Population Survey	2011–2013	2014–2018	Medicaid expansion in 2014	Non-expanded states
Fakeye, 2022	Administrative data from 2010—baseline year preceding Maryland Multi-Payor Patient-Centered Medical Home (PCMH) Program (MMPP) —till the final year of MMPP implementation (2013) on Medicaid insured and privately insured beneficiaries in Maryland	Second half of 2010	2011-2013	In April 2011, MMPP was established as a 3-year statewide demonstration of the efficacy of the PCMH model through which Medicaid and large private payers financially supported a group of primary care practices in the state of Maryland to function as advanced medical homes	A matched cohort of non-PCMH practices

eTable 4 (Cont.) Evidence Extraction Main Table

Study	Sample Size		Main findings	Conclusion	Funding source(s)
	Treatment group	Control group			

© 2024 Zhang et al. *JAMA Health Forum.*

Fischer, 2007	n = 19 (states); subgroup analysis (n = 4 treatment; n = 15 control)	n = 18 (states)	PA was associated with a 0.4% reduction (NS) in the proportion of RAAS-blocking-agent total units dispensed accounted for by ARBs and no effect on the subsequent trend in ARB use. In the subgroup analysis, PA with an ACE inhibitor requirement was associated with a statistically significant one-time decrease in ARB use of 1.6% ($P = 0.026$) after PA implementation, and a statistically significant decrease of 1.3% per calendar quarter ($P < 0.001$) on the trend in ARB use. PA with PDL approaches was associated with a slight but statistically significant increase of 0.5% per quarter ($P = 0.007$) in the slope of the trend in ARB use.	States that adopted PA in late 2004 based on PDL alone (only a subset of ARBs required prior authorization) saw an increase in ARB use after PA, compared with states having no PA. In the 4 states with ARB PA programs that required a prior trial of ACE inhibitors, ARBs use decreased after PA implementation.	Not reported
Maciejewski, 2010	n = 3545	n = 3545	Adherence to antihypertensive medications increased for exempt and nonexempt veterans in the year after the copayment increase (4.1% vs 5.9%; first difference −1.8%; 95% CI, −1.8 - −1.9) compared with the preperiod, but decreased thereafter for both groups compared with the preperiod. The decline in adherence to antihypertensive medications was greater for nonexempt veterans (−5.4% vs −2.3%; first difference −3.2%; 95% CI, −3.1 - −3.3).	Adherence to antihypertensive medications increased in the year after a $5 medication copayment increase but subsequently declined. The medication copayment increase had adverse effects on medication adherence.	Department of Veterans Affairs; one author was a consultant for Takeda Pharmaceuticals and owned stock in Amgen
Zhang, 2010	No-coverage group: n = 418; $150-cap group: n = 647; $350-cap group: n = 5093	n = 3027	The MPRs for any antihypertensive drug increased with coverage from 62.4% days covered in the no-coverage group to 81.1% in the $150-cap group, 82.7% in the $350-cap group, and 85.1% in the comparison group. The MPR in the comparison group did not change after introduction of Part D. Relative to the comparison group, intensity increased significantly in all 3 intervention groups, with the greatest increase in the no-coverage Part D group. Part D was associated with an increase of 0.22 (95% CI, 0.16-0.28) antihypertensive pills taken per day of treatment.	Medicare Part D was associated with improved medication adherence and increased treatment intensity for patients with hypertension. More generous coverage increased the intensity of medication treatment among beneficiaries using cardiovascular medications.	National Center for Research Resources; National Institute on Aging; one author was a director of and held equity in Aetna

© 2024 Zhang et al. *JAMA Health Forum.*

Zhang, 2011	No-coverage group: n = 1478; $150-cap group: n = 1326; $350-cap group: n = 8945	n = 4253	Antihypertensive use did not change $150-cap and $350-cap groups, but the proportion in the no-coverage group increased from 59.8% to 69.7% (OR 1.40, 95% CI, 1.25-1.56), and the increase is largest for ARBs. The proportion of ARB use appears to increase with the generosity of prescription coverage.	Transitioning from no drug coverage to Part D plans had increased odds of using any antihypertensive medication and increased counts of antihypertensive medications post-Part D. Part D likely translates into improved BP control.	National Institute of Mental Health; Agency for Healthcare Research and Quality; two authors had their research supported in part by Highmark Inc
Li, 2012	No gap coverage: n = 22 251; generic gap coverage: n = 3159; brand-name and generic gap coverage: n = 3709	n = 39 528	Patients with no gap coverage had an immediate decrease in the number of 30-day supply antihypertensive prescriptions in the month of gap entry, and the decrease continued in subsequent gap months, which translated into a decrease of 4.8% in the monthly number of antihypertensive prescriptions per patient. Monthly adherence decreased for patients with no gap coverage and generic-only gap coverage.	Part D coverage gap was associated with fewer monthly antihypertensive drug prescriptions, a higher likelihood of nonadherence, and continuous medication gaps among patients who had no gap coverage and generic-only gap coverage	Pfizer; American Heart Association
Wang, 2013	Pre-policy: n = 2525; post-policy: n = 2284	Pre-policy: n = 1700; post-policy: n = 1429	No significant difference was found in patients' persistence for antihypertensives between the 2 states in the pre- and post-policy period.	Monthly prescription restriction policy in the Louisiana Medicaid program was not significantly associated with the discontinuation of treatment for patients with hypertension	One author has received prior consulting support from Novartis and Takeda
Baicker, 2013	10,405 selected in the lottery (the lottery winners)	10,340 not selected (the control group)	Medicaid coverage did not have a significant effect on measures of blood pressure and no effect was found of Medicaid coverage on the use of medication for blood-pressure.	Medicaid coverage had no significant effect on the use of medication for hypertension.	Office of the Assistant Secretary for Planning and Evaluation, Department of Health and Human Services; the California HealthCare Foundation; the John D. and Catherine T. MacArthur Foundation; the National Institute on Aging (P30AG012810, RC2AGO36631, and R01AG0345151); the Robert Wood Johnson Foundation; the Alfred P. Sloan Foundation; the Smith Richardson Foundation; and the Social Security Administration (5 RRC 08098400-03-00, to the National Bureau of Economic Research as part of the Retirement Research Consortium of the Social Security Administration); and by the Centers for Medicare and Medicaid Services.

© 2024 Zhang et al. *JAMA Health Forum.*

Bardach, 2013	n = 42	n = 42	Intervention clinics had greater adjusted absolute improvement inblood pressure control (difference: 5.5% [95%CI, 1.6% to 9.3%], P = 0.01)	Pay-for-performance program in EHR-enabled small practices led to modest improvements in cardiovascular processes and outcomes.	Robin Hood Foundation, grants R18 HS17059 and R18 HS18275 from the Agency for Healthcare Research and Quality, K23 HD065836 from the National Institute for Children's Health and Human Development, and KL2 RR024130-05 from the National Center for Research Resources, the National Center for Advancing Translational Sciences, and National Institutes of Health, through the University of California San Francisco-Clinical and Translational Science Institute.
Petersen, 2013	(1) n=19 physicians; (2) n=20 physicians; (3) n=19 physicians	n=19 physicians	The adjusted estimated absolute difference between the proportion of the physician's patients achieving blood pressure control or receiving an appropriate response for the individual incentive group and the controls was 8.36% (95% CI, 2.40% to 13.00%; P = .005). the use of guideline-recommended no significant change in the use of guideline-recommended medication. The absolute difference in any medication adjustment (either to start a medication, add a medication, ormake a dose adjustment) was 15.36%(95%CI, 0.20% to 28.41%; P = .05) for the individual incentive group and control group.	Physicians who were randomized to the individual incentive group were more likely than controls to improve their treatment of hypertension as measured by achievement of blood pressure control or appropriate response to uncontrolled blood pressure. Those in the individual incentive group were more likely to make antihypertensive medication adjustments in response to uncontrolled blood pressures. The effect of the intervention was not sustained after the incentive was withdrawn.	Veterans Affairs (VA) Health Services Research & Development (HSR&D); Investigator-Initiated Research (IIR) program; National Institutes of Health; Robert Wood Johnson Foundation; American Heart Association Established Investigator Award
Zimmer, 2014	n = 15 133	n = 21 008	Number of medicines prescribed increased 11% per senior per year, for hypertension post-Part D, as compared to non-seniors	Part D has led to higher consumption of prescription drugs for hypertension patients	Not reported
Amin, 2017	n = 22 596	(1) n = 38 807; (2) n = 35 554	The fully adherent enrollees decreased adherence 1.2% (*P* < 0.001; 95% CI, −0.017 - −0.008) for patients with hypertension; the nonadherent and partially adherent treatment groups increased adherence after the policy changes by 0.7% (*P* = 0.28) and 0.5% (*P* = 0.27) in patients with hypertension	The cohort fully adherent to antihypertensives (PDC ≥ 80%) at baseline were the most affected by the policy changes, with a marked decrease in adherence as a result of the policy changes; those who were nonadherent to medication before the policy changes (PDC	Robert Wood Johnson Foundation and AcademyHealth

© 2024 Zhang et al. *JAMA Health Forum.*

				≤ 50%) showed no effect from the policy changes	
Cole, 2017	n = 492 (federally funded CHCs)	n = 565	Compared to centers in nonexpansion states in the pre- vs post-period, centers in expansion states had significant improvements in hypertension control (coef. 2.1, 95% CI, 0.2-4.0)	The first year of Medicaid expansion was associated with increases in BP control in states that expanded eligibility for Medicaid under the ACA	Agency for Healthcare Research and Quality
Hatch, 2017	n = 622	n = 622	Patients who gained Medicaid were significantly more likely to achieve a controlled hypertension measure (HR = 1.35, *P* < 0.001); average BPs were consistently lower but statistically insignificant for the group that gained Medicaid; a slightly larger but statistically insignificant proportion of patients who gained Medicaid received a prescription for medication compared to those who remained uninsured	Patients with uncontrolled hypertension who gained Medicaid coverage were more likely to achieve control within the study period	National Heart, Lung, and Blood Institute; Agency for Healthcare Research and Quality
McWilliams, 2017	n = 2 690 607 (24.8% of the total populations on antihypertensives)	n = 8 158 617	A slight differential increase in use of thiazides among beneficiaries with hypertension in the 2013 entry cohort (adjusted differential change, 0.5 percentage point; 95% CI, 0.1-0.8 percentage points; or 1.5% of the overall percentage using thiazides [33.4%]; *P* = .01). Slight differential increases in the PDC for β-blockers in the 2012 entry cohort (adjusted differential change, 0.3 percentage point; 95% CI, 0.1-0.5 percentage points; or 0.4% of the mean PDC [82.3%]; *P* = .003).	Through its 3rd year of operation, the MSSP has been associated with minimal increases in the use of or adherence to common antihypertensives	Laura and John Arnold Foundation; National Institute on Aging

© 2024 Zhang et al. *JAMA Health Forum.*

Kostova, 2017	n=1128	n=7872	Introduction of drug copayments in Medicaid was associated with an average rise in uncontrolled hypertension of 7.7 percentage points, but resulted in smaller and insignificant changes in the probability of taking antihypertensive drugs.	As Medicaid programs change in the years following the Affordable Care Act, prescription drug copayments may play a role as a lever for controlling hypertension at the population level.	Work from CDC. The authors declare no conflict of interest.
Adams, 2017	n = 196	n = 642	A marginally significant increase in antihypertensive days of supply in capped states (days' supply 8.60; 95 confidence intervals [CI]1.22 to 16.0) that was not present in non-capped states. A reversal in the white-black difference in trend for antihypertensive days supplied (trend change -1.83 days supplied per month; 95%CI -2.81 to -0.85).	Among dual enrollees with cancer, a modest increase in antihypertensive days' supply after transitioning to Medicare in capped states. The trends in antihypertensive use widened between white and blacks	National Institute on Aging (grant no. R01AG032249) and the Agency for Health Care Research and Quality (grant no. R01HS018577).
Hirth, 2017	n=64,165	n = 215,314	For people with heart disease, MPR for antihypertensive drugs did not change significantly. Among those with hypertension, the DID estimate between the two groups was 1 percentage point increase in MPR for ACE inhibitor medication in year 1 and year 2 after implementation, and was 0.8 percentage point increase in MPR for ARB medication in year 2, compared to baseline. Both estimates were statistically significant.	Adding enrollee participation requirements to value-based insurance design cost-sharing changes can maintain or slightly increase MPR between year 1, year 2 and baseline.	supported by the State government of Connecticut
Cole, 2018	n = 578 (CHCs)	n = 431 (CHCs)	CHCs in urban areas did not experience significant changes in hypertension control due to expansion; but in rural areas, Medicaid expansion was associated with relative improvements in 2015 in the percentages of BP control for patients with hypertension (2.1 percentage points; 95% CI, 0.2-4.0)	In rural areas, Medicaid expansion was associated with relative improvements in BP control in patients with hypertension; however, gains in quality due to expansion were not similarly observed in urban CHCs in expansion states	Agency for Healthcare Research and Quality

© 2024 Zhang et al. *JAMA Health Forum.*

Kaboli, 2018	group A: n = 143; group B: n = 128; group C: n = 131	n = 196	The unadjusted rate of thiazide prescribing was 9.7% for the control group (19 of 196) and 24.5% (35 of 143) for group A, 25.8% (33 of 128) for group B, and 32.8% (43 of 131) for group C (P < .001). Adjusted analyses demonstrated an intervention effect on thiazide prescribing at the index visit and 6-month visit, which diminished at the 12-month visit. For BP control, there was a significant intervention effect at the 12-month follow-up for group C (adjusted odds ratio, 1.73; 95%CI, 1.06-2.83; P = .04).	The patient activation intervention and a financial incentive and telephone call to the letter resulted in incremental improvements in thiazide prescribing. By 12 months, improved BP control was also evident.	The work reported here was supported by the Department of Veterans Affairs, Veterans Health Administration, Health Services Research and Development Service (IMV 04-066-1).
Diebold, 2018	n = 536 (n = 308 for a subgroup who maintained Part D coverage through 2010)	n = 1172	Part D appears to have increased the ordered log-odds that newly covered beneficiaries are in a higher, more beneficial, category of HBPUC by 0.59, but this effect was evident only in 2010	Part D enrollment did not appear to improve or prevent the progression of HBP among those previously diagnosed with hypertension. The observed health effects appear contingent upon continuous enrollment in Part D.	The author did not receive funding and has no conflicts of interest
Melissa, 2018	n = 160 (process incentive only)	n = 140 (control group)	The difference in BP under control between the process only incentive group and the control group was statistically significant (OR = 2.10, 95% CI, 1.01-4.35)	Financial incentives can improve uptake of prevention activities but modestly improve health	CMS under contract no. 500-00-1234
Angier, 2020	n = 3054 for newly insured; n = 12 314 for continuously insured patients	n = 2264 for continuously uninsured; n = 10 853 for discontinuously insured patients	Hypertension control rate for the newly insured increased 8.6% from 2012 to 2017; it increased only 0.9% for the continuously uninsured, increased 1.3% for the continuously insured, and increased 3.0% for the discontinuously insured group. In addition, the newly insured group living in the most deprived neighborhoods had a noticeable increase in hypertension control over the study period compared to the other insurance groups.	Hypertension control rates were largest among the newly insured from pre- to post-ACA Medicaid expansion, and the greatest increase in hypertension control occurred among newly insured patients living in the most deprived neighborhoods	Patient-Centered Outcomes Research Institute
Margerison, 2020	n = 16 499	n = 41 866	Medicaid expansion was associated with percentage point increases of 7.9 (95% CI, 3.1-12.8) in taking BP medication	Expanded Medicaid eligibility for low-income women of reproductive age was associated with increased health care utilization	Michigan Applied Public Policy Research pilot funding; Eunice Kennedy Shriver National Institute of Child Health and Human Development

© 2024 Zhang et al. *JAMA Health Forum.*

Marino, 2020	n = 2483 for newly insured; n = 5442 for continuously insured patients	n = 2888 for continuously uninsured; n = 5770 for discontinuously uninsured patients	The DiD estimate for adjusted mean SBP values: between the newly insured and continuously uninsured was 1.76 (95% CI, 1.34-2.19); between the newly insured and discontinuously uninsured was 1.23 (95% CI, 0.89-1.58); and between the newly insured and continuously insured was 1.49 (95% CI, 1.14 -1.84). The DiD estimate for adjusted mean DBP was 1.04 (95% CI, 0.77-1.30), 0.95 (95% CI, 0.73-1.17), and 1.00 (95% CI, 0.78 1.22), respectively.	Those who gained insurance following the ACA (newly insured) experienced greater improvements in BP levels as compared with the other insurance groups	National Heart, Lung, and Blood Institute; US Centers for Disease Control and Prevention, National Institute of Diabetes and Digestive and Kidney Diseases; Patient-Centered Outcomes Research Institute
Cole, 2021	n = 578 FQHCs, with 12 950 090 patients/year	n = 368 FQHCs, with 5 984 416 patients/year	Medicaid expansion was associated with a 1.61-PP increase (95% CI, 0.58-2.64) in the percentage of FQHC patients with hypertension with controlled BP, compared with FQHC patients in nonexpansion states, across the pooled 5-year expansion period. The association of expansion with BP control increased over time; by year 5, expansion was associated with a 2.36-PP (95% CI, 1.01-3.71) comparative increase in patients with controlled BP.	After 5 years of implementation, Medicaid expansion was associated with large, sustained improvements in BP control measures in a nationally representative sample of FQHCs	National Center for Advancing Translational Sciences of National Institutes of Health; National Institute on Drug Abuse; US Department of Defense; US Department of Veterans Affairs; Australian American Fulbright Commission; consulting fees from RAND Corporation
Gotanda, 2021	n = 4232	n = 1869	Medicaid expansions were associated with lower SBP (DiD estimate, − 3.03 mm Hg; 95% CI, −5.33 - − 0.73; *P* = 0.01; *P* = 0.03 after adjustment for multiple comparisons). DBP did not change after the Medicaid expansions.	Medicaid expansions were associated with a small reduction in SBP during the first 3 years of the program implementation, but there was no evidence that DBP changed significantly	No funding support and no conflict of interest
Kim, 2021	n = 17 states	n = 19 states	Medicaid expansion was associated with an additional 0.70% increase when the patients last took their BP medication	Medicaid expansion is significantly associated with increased health care utilization	No funding support and no conflict of interest
Lanese, 2021	n = 31 states	n = 20 states	BP control increased by 3.68% in expansion states (*P* = 0.0003)	BP control demonstrated a statistically significant improvement in expansion states vs non-expansion states	Akron Children's Hospital, Rebecca Considine Research Institute; no conflict of<br>interest is declared

© 2024 Zhang et al. *JAMA Health Forum.*

Peterson, 2021	n = 169 organizations (67 269 beneficiaries)<br>21 791 high CVD risk (32%)<br>45 478 medium CVD risk (68%)	n = 161 organizations (45 083 beneficiaries)<br>14 654 high CVD risk (33%)<br>30 429 medium CVD risk (67%)	Rates of initiation and intensification for antihypertensive medication were higher in the intervention vs control groups (2.5 percentage points; 95% CI, 0.9-4.2). Mean SBP was 1.2% lower in the intervention vs control groups.	Among patients at high risk with suboptimal risk factors, the Million Hearts Model increased the likelihood of initiating or intensifying antihypertensive medications by 5 percentage points (or 15%). The model effects were larger (in percentage terms) for initiation than for intensification. Among high-risk enrollees with follow-up data a mean of 13 months after enrollment, the model was also associated with roughly 1% reductions in SBP.	CMS
Sumarsono, 2021	n=29,541,647 pre-expansion; n=36,856,090 post-expansion	n=20,334,618 pre-expansion; n=21,877,889 post-expansion	Compared with the nonexpanded states, the Medicaid expanded states had a significantly greater increase in prescription rates of antihypertensives (DiD estimate [95% CI]: 63.2 [47.3–79.1], P<0.001).	The expansion of the Medicaid program in 2014 was associated with a significant increase in the use of evidence-based cardiovascular pharmacotherapies among Medicaid beneficiaries.	National Institutes of Health/National Center for Advancing Translational Sciences award UL 1TR-002541; National Heart, Lung, and Blood Institute (K23HL148525-1); National Institute of Aging GEMSSTAR Grant (1R03AG067960-01); National Institutes of Health (grant T-32HL125247-06).
Fakeye, 2022	n = 51 multi-payer PCMHs	n = 51 single-payer PCMHs; n = 42 non-PCMH practices	Among the privately insured patients, relative to single-payer PCMHs, multi-payer PCMHs saw a significantly lower decrease in MPR from baseline to the third (2.05% [0.72 - 3.37], *P* = 0.002) and fifth (1.67% [0.32 - 3.01], *P* = 0.02) half years of the demonstration; no significant differences were observed between multi-payer PCMHs and non-PCMH practices. Among Medicaid-insured patients, relative to multi-payer PCMHs, average MPR decreased significantly among single-payer PCMHs during the latter half of the demonstration, with the difference ranging between 3.81% and 6.04% during the final year of the demonstration, and no statistically significant differences were	Practices participating in a medical home demonstration financed by multiple private and public payers performed higher on medication adherence for their privately insured and Medicaid-insured patients than single-payer medical homes	No funding support and no conflict of interest

© 2024 Zhang et al. *JAMA Health Forum.*

	found in average MPR between non-PCMH practices and multi-payer PCMHs.	

Abbreviations: ARB, angiotensin II receptor blocker; RAAS, renin-angiotensin-aldosterone system; ACE, angiotensin-converting enzyme; VA, Veterans Affairs; MPR Medication Possession Ratio; DiD, difference-in-differences; PDC, proportion of days covered; CHC, community health center; HBPUC, high blood pressure under control; ACA, Affordable Care Act; SBP, systolic blood pressure; DBP, diastolic blood pressure; SE, standard error; HRSA, Health Resources and Services Administration; FQHCs, Federally Qualified Health Centers; CVD, cardiovascular disease; CMS, Centers for Medicare and Medicaid Services; PA, prior authorization; PDL, preferred drug list; EHR, electronic health record; ACO, Accountable Care Organizations; MSSP, Medicare Shared Savings Program; HRS, Health and Retirement Study; MIPCD, Medicaid Incentives for Prevention of Chronic Diseases; ADVANCE, Accelerating Data Value Across a National Community Health Center Network; CDRN, clinical data research network; PCMH, Multi-Payor Patient-Centered Medical Home; MMPP, Maryland Multi-Payor Patient-Centered Medical Home Program; NS, not significant; OR, odds ratio; HR, hazard ratio.

© 2024 Zhang et al. *JAMA Health Forum.*
