# Supplementary material for: Association of Economic Policies With Hypertension Management and Control: A Systematic Review
Source: JAMA Health Forum. 2024 Feb 9;5(2):e235231. doi: 10.1001/jamahealthforum.2023.5231 (PMC10858400; doi:10.1001/jamahealthforum.2023.5231)
Supplement: Supplement 2. — Data Sharing Statement [file jamahealthforum-e235231-s002.pdf]

Data Sharing Statement

Data
Data available: Yes
Data types: Data (not involving human participants)
How to access data: The detailed literature search results are included in the supplemental file.
When available: With publication

Supporting Documents
Document types: Other (please specify)
Additional Information: eTable 1. Search Terms and Definitions eTable 2. Inclusion and Exclusion Criteria eTable 3. Bias Assessment Checklist and Rubric eTable 4. Evidence Extraction Main Table
How to access documents: They are included in the supplemental file.
When available: With publication

Additional Information
Who can access the data: Anyone requesting the data
Types of analyses: Any purpose
Mechanisms of data availability: without investigator support
